# Impaired modulation of the trigeminal caudal nucleus by the locus coeruleus in diabetic mice: the role of GABAergic and glycinergic neurons

**DOI:** 10.3389/fnana.2025.1600026

**Published:** 2025-07-28

**Authors:** Alberto Mesa-Lombardo, Nuria García-Magro, Angel Nuñez, Yasmina B. Martin

**Affiliations:** ^1^Department of Anatomy, Histology and Neurosciences, Universidad Autónoma de Madrid, Madrid, Spain; ^2^Facultad de Ciencias de la Salud, Universidad Francisco de Vitoria, Pozuelo de Alarcón, Madrid, Spain; ^3^Facultad de Medicina, Universidad Francisco de Vitoria, Pozuelo de Alarcón, Madrid, Spain

**Keywords:** noradrenergic receptors, GABAergic neurons, glycinergic neurons, pain, diabetes

## Abstract

The trigeminal system plays a crucial role in processing somatosensory information, and its modulation is essential for maintaining sensory homeostasis. This study investigates the inhibitory modulation of trigeminal somatosensory responses by the locus coeruleus (LC), which is involved in numerous cognitive and physiological functions. Previous electrophysiological investigations have shown a diminished ability of the LC to inhibit somatosensory responses in the caudalis division of the spinal trigeminal nucleus (Sp5C) in diabetic mice. We hypothesize that *γ*-Amino-butyric acid (GABA)ergic and glycinergic neurons in the Sp5C may also participate in modulatory action from the LC. Using unit recordings in isoflurane anesthetized control and streptozotocin-induced diabetic mice, we examined the effect of LC electrical stimulation on GABAergic and glycinergic neurons, while monitoring trigeminal somatosensory responses to vibrissal stimulation. Local applications of bicuculline or strychnine (antagonists of GABAergic and glycinergic receptors, respectively) demonstrated that GABAergic and glycinergic neurons are under the control of noradrenergic projections from the LC by activation of α1 and α2 noradrenergic receptors, respectively. In diabetic mice, the functionality of these inhibitory circuits was reduced, contributing to the observed deficiency in LC-mediated sensory modulation and likely contributing to generation of neuropathic pain. Immunohistochemical experiments confirmed the presence of α1 and α2 noradrenergic receptors in GABAergic and glycinergic neurons that were altered in diabetic mice. In conclusion, the LC modulation exerted on the somatosensory responses in the Sp5C is not only through noradrenergic receptors on glutamatergic projection neurons but also exerts its control through projections to inhibitory interneurons.

## 1 Introduction

The locus coeruleus (LC) is a small, bilateral nucleus in the central nervous system (CNS) that plays a critical role in a wide range of cognitive and physiological functions (Glavin, 1985; Mitchell and Weinshenker, 2010; Borodovitsyna et al., 2017; Benarroch, 2018). LC neurons synthesize and release the neurotransmitter noradrenaline (NA), also known as norepinephrine, which is essential for modulating neuronal activity across various brain structures. These neurons extensively innervate sensory cortical and subcortical regions, and modulate neuronal activity (Foote et al., 1980; Loughlin et al., 1986; Berridge and Foote, 1991; Valentino et al., 1993; Waterhouse et al., 1998; Devilbiss and Waterhouse, 2004; Devilbiss et al., 2006; Hirata et al., 2006; Manella et al., 2017; Rho et al., 2018). The effect of NA depends on the types of receptors expressed on the postsynaptic neurons. In general, the activation of α1 or *β* NA receptors produces excitatory effects, whereas activation of α2 NA receptors induces inhibitory effects in various neuronal populations (Szabadi, 2013; Schwarz and Luo, 2015).

Furthermore, the LC plays an important role in pain perception (Brightwell and Taylor, 2009; Donertas-Ayaz and Caudle, 2023). Neuropathic pain increases spontaneous and noxious-evoked activity of LC neurons (Alba-Delgado et al., 2021). In addition, the descending NA inhibitory system on dorsal horn and trigeminal neurons is one of the main pathways involved in the endogenous pain modulation system (Pertovaara, 2013; Llorca-Torralba et al., 2016). Previous experiments showed that stimulation of the LC increases the content of NA in the spinal cord (Crawley et al., 1979) and inhibits nociceptive responses in the dorsal horn neurons by activation of α2 NA receptors (Hodge et al., 1981; Jones and Gebhart, 1986; Jones, 1991; West et al., 1993). Electrical stimulation of the LC also inhibits sensory responses in the trigeminal complex (McBride and Sutin, 1984; Mesa-Lombardo et al., 2023). Moreover, it has been reported that specific stimulation of NA neurons in the LC by optogenetic techniques produces a significant antinociceptive effect in the thermal hind paw withdrawal test (Hickey et al., 2014), demonstrating that these neurons are responsible for the control of pain perception. Therefore, LC stimulation is capable of relieving neuropathic pain by attenuating allodynia and hyperalgesia through the increased release of NA (Pertovaara, 2013).

The caudalis division of the spinal trigeminal nucleus (Sp5C) receives extensive afferent innervation from peripheral sensory neurons and is the first central relay in the circuitry involved in processing orofacial pain and non-noxious sensory stimuli from the craniofacial region (Jacquin et al., 1986; Hu, 1990; Bae et al., 2000; Bereiter et al., 2000; Bae et al., 2003; Shinoda et al., 2019; Terrier et al., 2022). It has been proposed that local inhibitory circuits involving *γ*-Amino-butyric acid (GABA) ergic and/or glycinergic interneurons may modulate neuronal responses in the sensory trigeminal nuclei (Zarbin et al., 1981; Jacquin et al., 1989; Rampon et al., 1996; Ressot et al., 2001; Avendaño et al., 2005; Bae et al., 2005). Anatomical evidence indicates that the LC sends NA projections to the sensory trigeminal nucleus (Levitt and Moore, 1979; Couto et al., 2006); however, the mechanisms that control trigeminal activity remain poorly understood. Electrophysiological studies have shown that the spontaneous neuronal activity and responses of Sp5C neurons to tactile or nociceptive inputs are inhibited by the LC (Sasa and Takaori, 1973; Baba et al., 2000; Tsuruoka et al., 2003b; Mesa-Lombardo et al., 2023). Nociceptive information in the orofacial area is conveyed principally to the Sp5C through the trigeminal nerve (Marfurt and Turner, 1984; Bae et al., 2003; Takemura et al., 2006), most of whose terminals are located in laminae I and II (Priestley et al., 1982; Jacquin et al., 1986; Wang et al., 2000). Lamina I neurons are glutamatergic and transmit sensory information to higher brain regions. Lamina II neurons respond to sensory stimuli, and most of them are GABA/glycinergic interneurons projecting to lamina I (Basbaum et al., 1986; Jacquin et al., 1989; Wang et al., 2000). In addition, laminae III-IV receives non-noxious sensory information.

Neurons in laminae I and II receive dense modulatory projections from higher brain regions, such as NA inputs from the LC and serotonergic inputs from raphe nuclei, in addition to nociceptive inputs from the peripheral nervous system. NA fibers are also present in laminae III-IV (Dickenson et al., 1981; Cropper et al., 1984; Tsuruoka and Willis, 1996; Tsuruoka et al., 2003a). The Sp5C is considered to play a critical role in the regulation of nociception in the trigeminal nervous system. Most of NA effects on Sp5C neurons are mediated by activation of α2-NA receptors (Donertas-Ayaz and Caudle, 2023). However, there is limited evidence regarding the effect of NA inputs on inhibitory neurons in the Sp5C nucleus and their participation in sensory processing, particularly in nociception.

GABA is well known as an inhibitory amino acid neurotransmitter in the CNS and may modulate nociceptive responses in the spinal cord (Sivakumar and Ramli, 2022) and in the trigeminal complex, mainly through the activation of GABA_A_ receptors (Storer et al., 2001; Garcia-Magro et al., 2020; Garcia-Magro et al., 2021). Glycinergic transmission has also been described in the spinal cord and in the trigeminal complex (Zeilhofer et al., 2021). Immunohistochemical studies have demonstrated the presence of a high density of glycine receptors and glycinergic neurons in the Sp5C that inhibit sensory responses (Zarbin et al., 1981; Rampon et al., 1996).

Diabetic neuropathy is a common complication of both type 1 and type 2 diabetes that results in sensory loss and pain. One of the most important reasons for the generation of peripheral neuropathy may be the impairment of neuronal activity caused by diabetes. Lesioned sensory neurons, as occurs in diabetic neuropathy, develop hyperexcitability, and thus can generate action potential discharges in the absence of stimuli and exhibit an altered stimulus–response function (Suzuki et al., 2000; Orstavik et al., 2006). Therefore, these findings suggest that chronic pain might emerge from an imbalance between activation (nociceptive inputs) and inhibition (via NA, serotonin or dopamine); (España et al., 2024). We have demonstrated previously that the inhibitory influence of the LC on Sp5C vibrissal responses is reduced in a mouse model of diabetes (Mesa-Lombardo et al., 2023). Taking into account that the LC modulates tactile and nociceptive responses in the Sp5C nucleus and that GABAergic and glycinergic inhibitory neurons are important in the control of sensory processing in this relay station of the somatosensory pathway, the aim of the present study is to determine the effect of the LC on these inhibitory neurons and how their alteration in diabetes may be responsible for the generation of neuropathic pain in these patients. We have performed unit recordings in anesthetized mice and immunohistochemical studies to reveal NA modulation of Sp5C inhibitory neurons in control and in streptozotocin (STZ)-diabetic mice.

## 2 Materials and methods

The experiments were performed using young adult (2–3 month old) male C57BL/6 J mice (*N* = 84; weight ranged between 22 and 28 g. Harlan Laboratories, Spain). The mice were divided into two groups, STZ-induced diabetic mice (*N* = 36) and control mice (*N* = 48) that only received vehicle. All mice were housed under a 12:12-h dark/light cycle at 22 ± 2°C with food and water ad libitum. All the animal procedures followed the European guidelines (2010/63, European Council Directives) and were approved by the local Ethical Committee (Autonomous University of Madrid and Government of the Community of Madrid; PROEX: 181.6/21). Efforts were made to minimize animal suffering as well as to reduce the number of mice used.

### 2.1 STZ-dependent diabetes

STZ is an antibiotic that produces pancreatic islet *β*-cell destruction and is widely used experimentally to produce a model of type 1 diabetes mellitus (Furman, 2021). This animal model is employed for assessing the pathological consequences of diabetes and for screening potential therapies for the treatment of this condition. STZ (50 mg/kg, intraperitoneally, i.p.; Sigma-Aldrich, St. Louis, MO) was administered for 5 consecutive days. The mice were considered diabetic when their glucose levels in the tail blood after a 4-h fast were >300 mg/dL (glucometer: Glucoleader-Yasee GLM-76, Nessler, Spain). The control mice were injected with the vehicle (10 mM sodium citrate, 0.9% NaCl; pH 4.5, i.p.). Glucose measurements were performed prior to the STZ injection, and throughout the 3 weeks diabetes development, as well as before the experimental recordings, or before each of the behavioral tests.

### 2.2 Behavioral test

An evaluation of the response to mechanical allodynia was conducted using a set of six calibrated nylon von Frey monofilaments with bending forces ranging from 0.008 to 0.4 g to verify that all diabetic mice used in the rest of the experiments experienced pain in the orofacial area. The procedure was performed using the protocol described by Mesa-Lombardo et al. (2023). Briefly, von Frey filaments were applied in ascending order five times on five different points of each vibrissal pad. Each probe was applied until it just bent. The time interval between consecutive filament administrations was at least 5 s. The response threshold was considered as the lowest force of the filaments that produced a brisk head withdrawal in more than 50% of trials (3 out of 5). This test was performed two and three weeks after STZ or vehicle injection.

### 2.3 Unit recordings and tactile stimulation

After the 3 weeks of diabetes development in STZ-injected animals or 3 weeks of receiving vehicle in control animals, animals were recorded in the next week. The mouse was anesthetized with isoflurane (2% induction; 1–1.5% maintenance doses) and placed in a David Kopf stereotaxic apparatus (Tujunga, CA, USA). Body temperature was set at 37°C through a water-heated pad (Gaymar T/Pump, Orchard Park, NY, USA). The skin over the midline of the scalp was sectioned and retracted. A small craniotomy was drilled over the LC nucleus according to the atlas of Paxinos and Franklin (coordinates from Bregma: A: −5.4 mm, L: 0.9 mm lateral, H: 3.5 mm) (Paxinos and Franklin, 2003).

Tungsten microelectrodes (2 MΩ; AM-System, Sequim, USA) were used to obtain single unit recordings in the Sp5C (A: −7.6 mm, L: 2 mm from bregma; H: 0.5–1.5 mm from the surface of the nucleus). The recording electrode was introduced at a 60° angle to the surface of the nucleus after opening the cisterna magna. The position of the electrodes was visually controlled under a dissecting microscope. Unit recordings were filtered between 0.3–3 kHz and amplified using a DAM50 preamplifier (World Precision Instruments, Friedberg, Germany). The signals were sampled at 10 kHz through an analog-to-digital converter (Power 1,401 data acquisition unit, Cambridge Electronic Design, Cambridge, UK) and fed into a PC for off-line analysis with Spike 2 software (Cambridge Electronic Design).

Vibrissal deflections were evoked by brief air-pulses using a pneumatic pressure pump (Picospritzer, Hollis, NH, USA; 1–2 kg/cm^2^, 20 ms duration), delivered through a 1 mm inner diameter polyethylene tube. The experimental protocol consisted of air pulses delivered to the vibrissae at 0.5 Hz for 1 min (30 stimuli; basal condition) before electrical pulses in the LC nucleus followed by vibrissal stimuli at 50–300 ms delays for 1 min (30 pair of stimuli; pair pulses). After the paired-pulse protocol, the vibrissal was stimulated for 1 min at 0.5 Hz (30 stimuli) to test if the vibrissal response recovered from the effect of LC stimulation. In one of the experiments, we applied pair pulses of stimuli at the vibrissa with a short delay (50, 100 and 300 ms) to quantify feedback inhibition in the Sp5C nucleus.

LC electrical stimulation was performed by a bipolar stimulation electrode (World Precision Instruments, Friedberg, Germany) aimed at the LC nucleus (A: −5.4 mm, L: 0.9 mm lateral, H: 3.5 mm). Pulses of 0.3 ms duration and 10–100 μA intensity were applied. For comparison, the stimulation intensity was set two times higher than the threshold to elicit spike firing in the Sp5C neurons.

### 2.4 Drugs

The antagonist of *α*2-NA receptors yohimbine (2 mg/Kg), the non-selective α2-NA agonist clonidine (2 mg/Kg) was i.p. administrated 30 min before the recording session. The GABA_A_ receptor antagonist bicuculline methiodide (20 mM), the α1-NA antagonist benoxathian (20 μM) and the glycinergic receptor antagonist strychnine (100 μM) were locally injected in the Sp5C nucleus with a canula attached to 1 μL Hamilton syringe. The α-amino-3-hydroxy-5-methyl-4-isoxazolepropionic acid (AMPA) receptor antagonist 6-Cyano-7-nitroquinoxaline-2,3-dione, 6-Cyano-2,3-dihydroxy-7-nitro-quinoxaline (CNQX) was also locally injected. All drugs (Sigma, St Louis, MO, USA) were dissolved in saline solution (0.9% NaCl). The injected volume was 0.2 μL. Recordings were performed after drug application, with the same protocol as in basal conditions.

### 2.5 Immunohistochemistry

Young adult male mice C57BL6 were deeply anesthetized (Dolethal, 50 mg/kg i.p. Vétoquinol; Madrid, Spain) and perfused through the ascending aorta with saline 0.9% followed by 4% paraformaldehyde in 0.1 M phosphate buffer (PB; pH 7.4). The brainstem was extracted and postfixed in the same fixative overnight and then cryoprotected for 3 days in 30% sucrose in PB 0.1 M. The block of brainstem were frozen and coronal sections of 30-μm-thick were cut serially using a sliding microtome (Leica SM2400, Leica Biosystems, Nussloch) and collected in PB.

The sections were incubated free-floating in blocking solution (PB 0.1 M, 1% Triton X-100 and donkey normal serum (DNS) 10%) for 2 h at room temperature, followed by incubation at 4°C with different combinations of primary antibodies: rabbit anti-α1 (1:100; A270, Sigma Aldrich), goat anti-α2 (1:100; PAB6968, Abnova), mouse anti-gad67 (1:800; MAB5406, Thermo Fisher), rabbit anti-glycine (1:100: ab9443, Abcam) and guinea pig anti-VGLUT2 (1:2000: MAB5504, Sigma-Aldrich) in a blocking solution for 24 h. After several washing in PB 0.1 M, sections were incubated with a mix of polyclonal secondary antibody donkey anti-rabbit AlexaFluor 488 (1:200, A21206, ThermoFisher), donkey anti-mouse AlexaFluor 546 (1:200; A10036, ThermoFisher), donkey anti-guinea pig AlexaFluor 633 (1:200; SAB4600129, Sigma-Aldrich), donkey anti-mouse, AlexaFluor 647 (1:200, A31571, ThermoFisher) and donkey anti-goat AlexaFluor 546 (1:200; A11056, ThermoFisher) for 2 h in the dark at room temperature. In addition, after 2 washes in PB, all nuclei were labeled with a dilution of Bisbenzimide (Hoescht 1:3000) in PB (Table 1). Finally, all sections were mounted on glass slides and coverslips with ProlongTM (Thermo Fisher Scientific, Waltham, MA, USA).

**Table 1:** Primary and secondary antibodies.

Antigen and host species	Dilution	Product code and source
Primary antibodies		
Anti-GAD67, mouse monoclonal	1:800	MAB5406, Sigma-Aldrich
Anti-Glycine, rabbit polyclonal	1:100	ab9442, Abcam
Anti-α1-AR, rabbit polyclonal	1:100	A270, Sigma-Aldrich
Anti-α2-AR, goat polyclonal	1:100	PAB6968, Abnova
Anti-vGLUT2, guinea pig polyclonal	1:2000	MAB5504, Sigma-Aldrich
Secondary antibodies		
Donkey anti-guinea pig, CF 633	1:200	SAB4600129, Sigma-Aldrich
Donkey anti-goat, AlexaFluor 546	1:200	A11056, ThermoFisher (Invitrogen)
Donkey anti-mouse, AlexaFluor 546	1:200	A10036, ThermoFisher (Invitrogen)
Donkey anti-mouse, AlexaFluor 647	1:200	A31571, ThermoFisher (Invitrogen)
Donkey anti-rabbit, AlexaFluor 488	1:200	A21206, ThermoFisher (Invitrogen)

### 2.6 Microscopy and image analysis

Confocal microscopy 3D images of the Sp5C in both sides were obtained using a TCS SP5 Spectral Leica confocal microscope (Leica Mycrosystems AG; Wetzlar, Germany) using a 63X, 40X or 20X oil immersion objectives to study the morphology and location of Sp5C neurons or the densitometry analysis, respectively. Image stacks were acquired at 1024 × 1,024 pixels using Leica LAS AF software.

The analysis was carried out using the ImageJ image analysis software for Windows (Microsoft; Albuquerque, NM, USA). Images obtained by confocal microscopy were processed to create TIFF files with maximum intensity projections, using a consistent final tissue thickness of 10 μm for all series. To ensure comparable immunostaining, sections were processed together under identical conditions, and a threshold was set to eliminate background. The region of interest (ROI) was delineated to include the Sp5C, two broad ROIs were defined to include lamina II and laminae III-IV. A densitometric analysis of immunoreactivity was performed on each image, obtaining optical density measurements using the ImageJ ‘Set Measurement’ routine. These gray values were used to generate histograms and perform statistical analysis.

### 2.7 Statistical analysis

The peristimulus time histograms (PSTHs) were used to calculate spike responses in a 50 ms post-stimulus time window following each stimulus (1 ms bin-width). The mean response during the basal recording was considered to be 100% and the effect of LC stimulation during the paired-pulse stimulation protocol was calculated. In the experiment of paired-pulse stimulation of the vibrissa, the mean response to the first stimuli was considered 100% and the reduction of to the second response was considered as a measure of the feedback inhibition.

Statistical analysis was performed using Graph Pad Prism 10 software (San Diego, CA, USA). Any differences between the variables were compared using two-way parametric (Student’s t test or paired t-test) after normality testing (Kolmogorov–Smirnov normality test). The sample size for each experiment was chosen based on previous experience. Data were expressed as the mean ± standard error of the mean (SEM) with n indicating the number of neurons analyzed or N the number of mice per group for a given experiment. All data were collected from a minimum of four mice per experiment. The results were considered significant at *p* < 0.05 (*p < 0.05, **p < 0.01, ***p < 0.001).

## 3 Results

**Figure 1:**
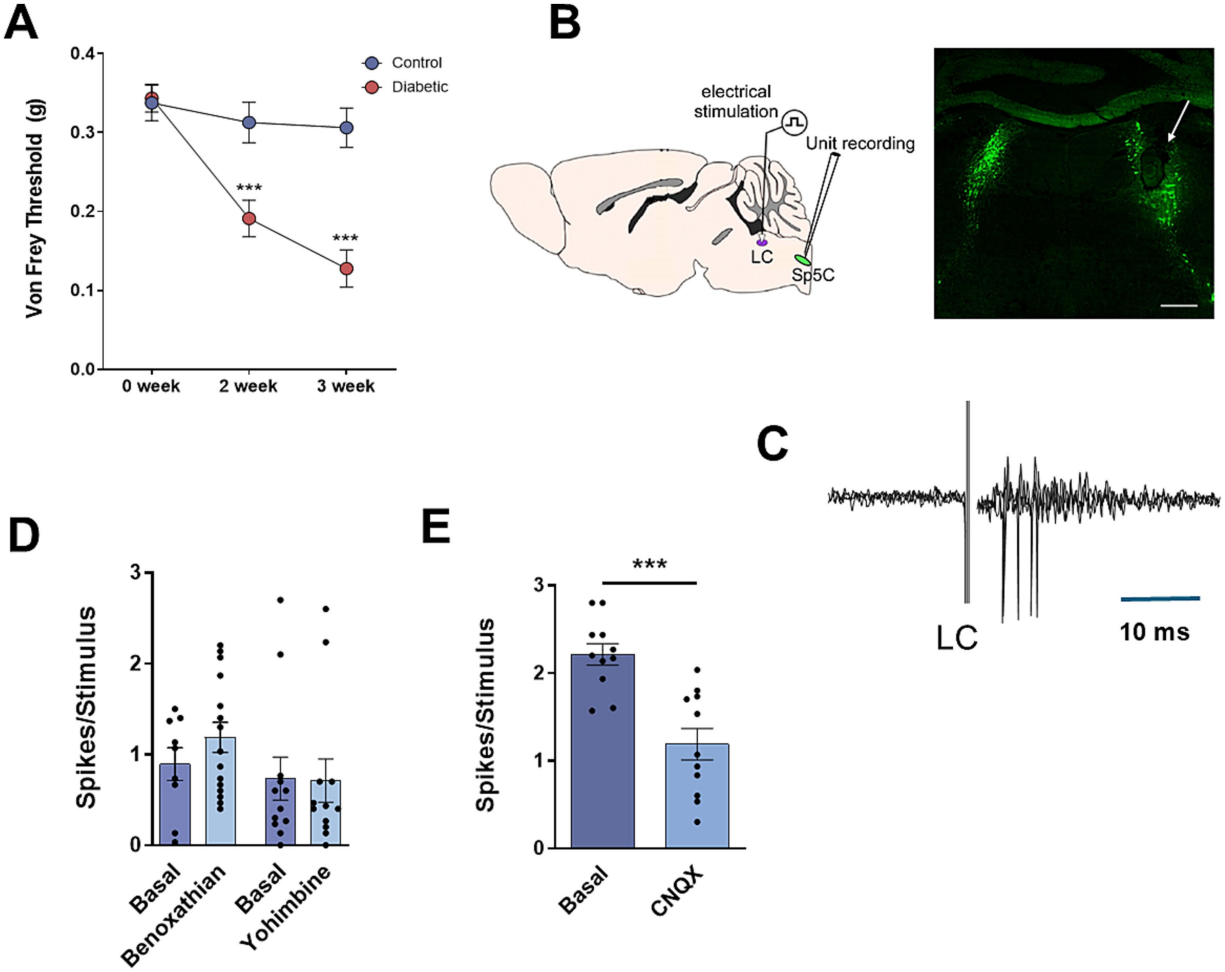
LC induces orthodromic responses in Sp5C. **(A)** Diabetic animals show a decrease in the withdrawal threshold to mechanical stimulation in vibrissal pad the second and third week after STZ injection in comparison with basal values obtained before STZ injection. **(B)** Schematic diagram of the experimental setup. Location of the bipolar electrode is shown in a representative photomicrograph of tyrosine hydroxylase positive (TH+) labeled cells in the LC of a control mouse (inset; white arrow; Scale 400 μm). **(C)** LC electrical stimulation (0.3 ms; 20 μA; 0.5 Hz) induces orthodromic responses in a representative Sp5C neuron recorded in a control mouse; three superimposed traces are shown. **(D)** The orthodromic response is not altered by the α1 NA receptor antagonist benoxathian or the α2 NA receptor antagonist yohimbine (20 μM; 0.2 μL). **(E)** Local application of the AMPA receptor antagonist CNQX (20 nM; 0.2 μL) reduces orthodromic responses, suggesting that it is meanly mediated by glutamatergic receptors. In this and in the following figures *p < 0.05; **p < 0.01; ***p < 0.001.

We have studied the modulatory effect of the LC on vibrissal responses in the Sp5C nucleus in STZ-diabetic mice and in control mice. We have performed the experiments when the animals showed a glucose level higher than 300 mg/dL (484.5 ± 15.5 mg/dL; *N* = 36) and a reduced pain threshold, 3 weeks after STZ injection. To test the presence of pain, the Von Frey withdrawal threshold to mechanical stimulation of the vibrissal pad was measured two and three weeks after STZ or vehicle injection. In control animals we observed a slight reduction of the threshold with time (0.33 ± 0.022 g; 0.31 ± 0.03 g; 0.31 ± 0.03 g; basal values, 2 weeks and 3 weeks after vehicle injection; *p* > 0.05; *N* = 48; Figure 1A). However, the withdrawal threshold decreased faster in diabetic animals (0.34 ± 0.02 g; 0.1 9 ± 0.02 g; 0.13 ± 0.02 g; basal values, 2 weeks and 3 weeks after STZ injection; *n* = 36; *p* < 0.0001 in both cases respect to basal values; Figure 1A). STZ-diabetic animals that did not show a significant decrease in withdrawal threshold at 3 weeks were not included in this study.

### 3.1 LC modulates the activity of Sp5C neurons

To test the modulatory effect of LC on non-nociceptive tactile stimuli delivered in the vibrissal pad, unit recordings were performed in Sp5C, and electrical stimuli were applied to the LC (Figure 1B). Vibrissal stimulation evoked orthodromic responses in both control and diabetic mice (2.5 ± 0.1 spikes/stimulus; *n* = 37 neurons and 2.3 ± 0.4 spikes/stimulus; *n* = 25 neurons, respectively; *p* > 0.05; unpaired test). In addition, electrical stimulation of the LC (0.3 ms duration; 10–100 μA intensity) induced a short latency orthodromic response in the Sp5C neurons in control and in STZ-diabetic mice. The orthodromic response consisted in 1–2 spikes with a short latency (6.1 ± 0.5 ms; 1.6 ± 0.18 spikes/stimulus; *n* = 75 neurons and 6.5 ± 0.3 ms; 1.1 ± 0.2 spikes/stimulus; *n* = 63 neurons, control and STZ-diabetic mice, respectively; *p* > 0.05; unpaired test) that was followed by a decrease of the spontaneous firing (Figure 1C). To test the origin of this orthodromic response the α1-NA receptor antagonist benoxathian or the α2-NA receptor antagonist yohimbine were locally applied (20 μM; 0.2 μL). In control animals, the orthodromic response to LC stimulation was slightly increased by benoxathian but differences were not statistically significant (0.9 ± 0.18 basal values; 1.2 ± 0.16 spikes/stimulus; *n* = 15 neurons; *p* > 0.05; paired test; Figure 1D). Therefore, yohimbine did not alter LC response (0.7 ± 0.23 basal values; 0.7 ± 0.24 spikes/stimulus; *n* = 12 neurons; *p* > 0.05; paired test), suggesting that LC orthodromic response was not due to activation of NA receptors. However, the orthodromic response was significantly reduced by local application of AMPA receptor antagonist CNQX (20 nM; 0.2 μL; 2.2 ± 0.12 basal values; 1.2 ± 0.18 spikes/stimulus; *n* = 11 neurons; *p* = 0.0003; Figure 1E), indicating that it was due to activation of glutamatergic receptors.

**Figure 2:**
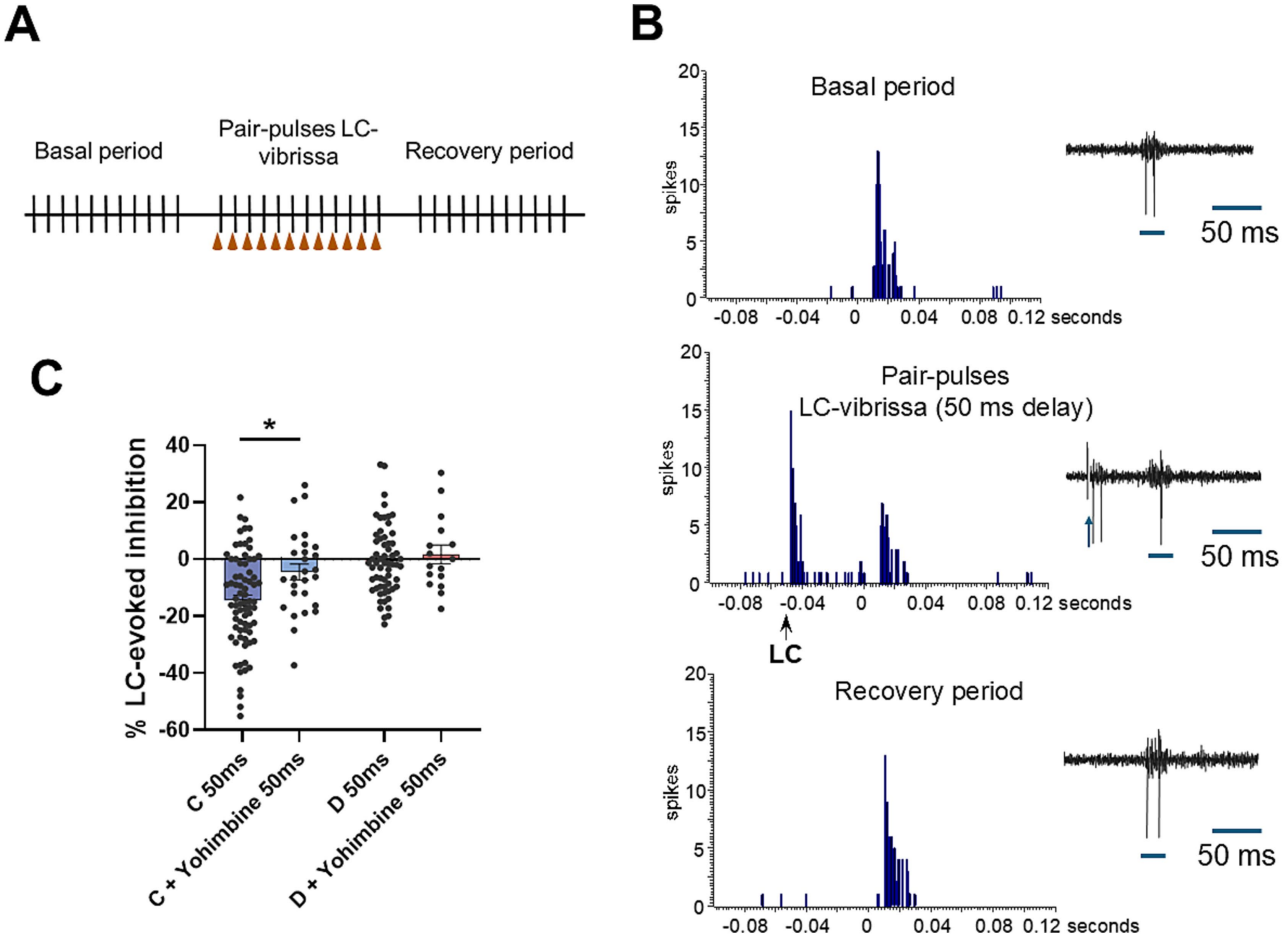
LC stimulation induces an inhibition of vibrissal responses through activation of α2-NA receptors that is reduced in STZ-diabetic mice. **(A)** Stimulation protocol to test the effect of LC stimulation is shown. **(B)** Representative PSTHs during the basal period (upper histogram), when LC and vibrissal stimuli are paired (50 ms delay between LC and vibrissal stimuli; middle histogram) and during the recovery period (lower histogram) in a control mouse. Vibrissal response is reduced by LC stimulation. **(C)** Plot shows the mean LC-evoked inhibition in control (C, blue) and in STZ-diabetic (D, red) mice in basal conditions and after i.p. injection of yohimbine, antagonist of α2-NA receptors (2 mg/Kg; i.p.). Note that LC-evoked inhibition is lower in STZ-diabetic mice respect to control mice. Yohimbine reduces LC-evoked inhibition in control animals but does not affect STZ-diabetic animals.

To study the role of LC in the modulation of sensory transmission, we focused our experiments on the inhibitory action of LC. Electrical stimulation of the LC reduced vibrissal responses when they were paired with a short delay (50 ms, Figures 2A,B), as has been described previously (Sasa and Takaori, 1973; Baba et al., 2000; Tsuruoka et al., 2003b; Mesa-Lombardo et al., 2023). LC stimulation induced a reduction of vibrissal responses of 14.5 ± 1.9% in control animals (*n* = 41 neurons; Figure 2C). This reduction was not observed in STZ-diabetic mice (0.6 ± 1.6%; *n* = 36 neurons; *p* < 0.0001, respect to control animals). We termed this response reduction as LC-evoked inhibition. It was decreased by the α2-NA receptors antagonist yohimbine (2 mg/Kg; i.p.) up to 4.5 ± 2.8%; (*n* = 27 neurons; *p* = 0.0232) in control animals. The LC-evoked inhibition in STZ-diabetic mice was not affected (1.7 ± 3.2%; *n* = 16; *p* > 0.05; Figure 2C).

**Figure 3:**
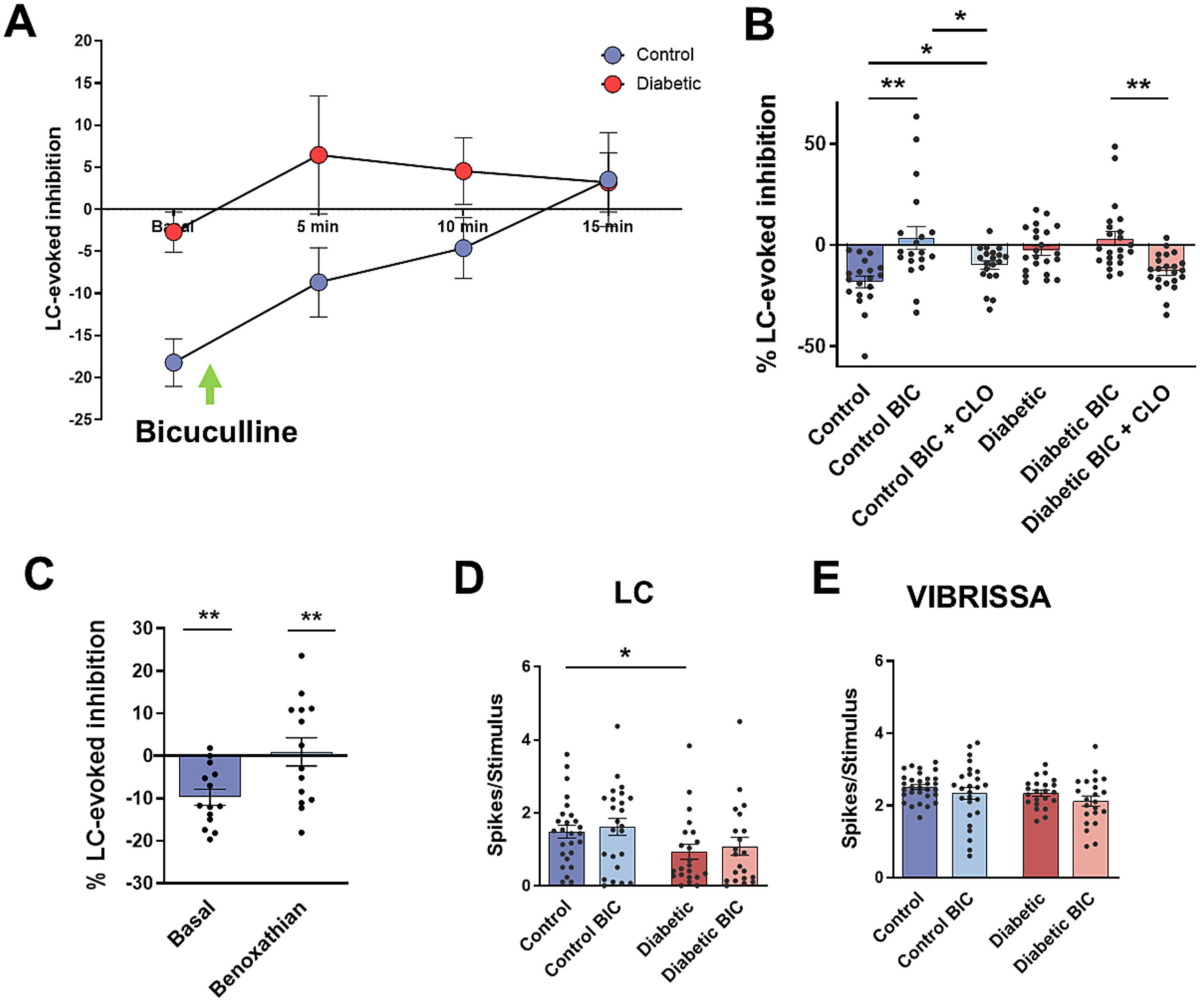
Bicuculline blocks LC-evoked inhibition. **(A)** Time course of the change in the LC-evoked inhibition in basal condition and 5, 10, 15 min after local injection of the GABA_A_ receptor antagonist bicuculline (BIC; 20 mM, 0.2 μL). LC-evoked inhibition is gradually reduced in control mice. In STZ-diabetic mice, the small LC-inhibition is transformed in a facilitation 5 min after bicuculline injection and remained equal for 15 min. **(B)** Plot showing the mean value of LC-evoked inhibition in basal conditions and 15 min after BIC injection. LC-evoked inhibition is blocked in control mice; however, BIC does not affect the inhibition in STZ-diabetic mice. In this condition, the α2-NA receptors agonist clonidine increases LC-evoked inhibition in both animal groups. **(C)** Plot of the mean LC-evoked inhibition in basal condition and after local injection of α1-NA receptor benoxathian (20 μM; 0.2 μL). LC-evoked inhibition is blocked indicating that is due to activation of α1-NA receptors. **(D)** Plot of the mean response to LC stimulation. The response is lower in STZ-diabetic mice. However, bicuculline does not affect the response. **(E)** Plot of the mean response to vibrissal stimulation. Bicuculline has not effect in the response.

Moreover, LC-evoked inhibition was blocked by local application of the GABA_A_ receptor antagonist bicuculline in control mice (20 mM, 0.2 μL; Figure 3A). Bicuculline reduced progressively LC-evoked inhibition at 5–10 min after application, reaching a complete block at 15 min. In STZ-diabetic mice the small LC-evoked inhibition in basal conditions was converted in a facilitation 5 min after bicuculline injection. Figure 3B shows mean value of LC-evoked inhibition in basal conditions and 15 min after bicuculline injection. In control animals, the LC-evoked inhibition reduced vibrissal responses by 18.2 ± 2.2% in basal conditions, while 15 min after bicuculline the LC-evoked inhibition disappeared (a slight facilitation of 3.6 ± 4.7%; *p* = 0.004; *n* = 19 neurons; Figure 3B). In STZ-diabetic mice, the small LC-evoked inhibition observed in basal conditions (2.7 ± 2.4%) was replaced by a facilitation of vibrissal responses after bicuculline local injection (3.18 ± 2.8%; *p* > 0.05; *n* = 22 neurons).

In these conditions when LC-evoked inhibition was blocked by bicuculline, α2-NA receptors remained active because of i.p. injection of clonidine increased LC-evoked inhibition in either control and STZ-diabetic animals (9.3 ± 1.9%; *p* = 0.0259; *n* = 20 neurons and 13.1 ± 2.8%; *p* = 0.0006; *n* = 22 neurons, respectively; Figure 3B), indicating that these receptors were not located in GABAergic neurons.

The above results indicated that GABAergic neurons were partially responsible of the LC-evoked inhibition of Sp5C. Considering that α2-NA receptor activation induces inhibition in most of the neurons studied (Szabadi, 2013; Schwarz and Luo, 2015) and that α2-NA receptors were not located in GABAergic neurons, we proposed that the α1-NA receptor could be responsible for the activation of GABAergic neurons. Thus, we tested the effect of the α1-NA receptor antagonist benoxathian on the LC-evoked inhibition. Local injection of benoxathian (20 μM; 0.2 μL) reduced LC-evoked inhibition from 9.8 ± 2.2% to 0.1 ± 4.6% (*p* = 0.0088; *n* = 15 neurons; Figure 3C), indicating that it was partially due to GABAergic neurons through activation of α1-NA receptors.

Contrary to expectations, orthodromic responses from LC stimulation were not affected by bicuculline in control animals (1.48 ± 0.18 spikes/stimulus in basal conditions and 1.61 ± 0.21 spikes/stimulus after the injection of bicuculline; *p* > 0.05; *n* = 19 neurons; Figure 3D). In STZ-diabetic mice LC stimulation elicited a smaller response respect to control mice (0.93 ± 0.2 spikes/stimulus; *p* = 0.0478; *n* = 22 neurons, respect to control values) that increased 10 min after the injection of bicuculline (1.47 ± 0.47 spikes/stimulus; *p* > 0.05; *n* = 22 neurons). The bicuculline did not also affect vibrissal responses in both control and STZ-diabetic mice. In control animals, vibrissal stimulation evoked 2.5 ± 0.07 spikes/stimulus in basal conditions and 2.4 ± 0.23 spikes/stimulus 15 min after the injection of bicuculline (*p* > 0.05; *n* = 19 neurons). In STZ-diabetic animals, vibrissal stimulation induced 2.3 ± 0.08 spikes/stimulus in basal conditions and 2.1 ± 0.18 spikes/stimulus 15 min after the injection of bicuculline in STZ-diabetic animals (*p* > 0.05; *n* = 22 neurons; Figure 3E).

### 3.2 LC-evoked inhibition was also modulated by glycinergic neurons

**Figure 4:**
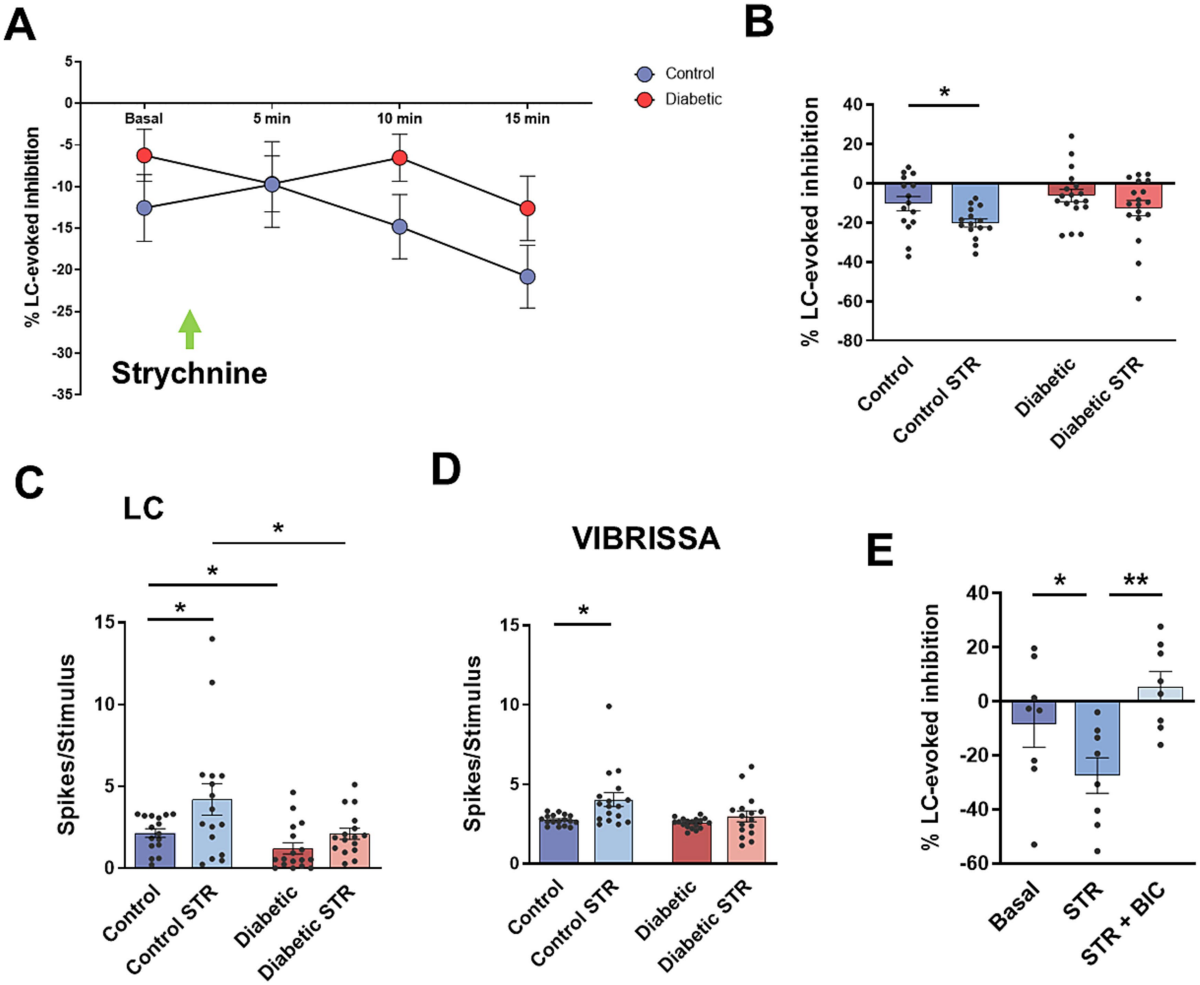
Strychnine increases LC-evoked inhibition. **(A)** Time course of the change in the LC-evoked inhibition in basal condition and 5, 10, 15 min after local injection of the glycinergic receptor antagonist strychnine (STR; 100 μM; 0.2 μL). LC-inhibition increases after strychnine injection, both in control and STZ-diabetic mice. **(B)** Plot showing the mean value of LC-evoked inhibition (50 ms delay) in basal conditions and 15 min after STR injection. LC-evoked inhibition increases reaching statistical significance in control mice. **(C)** Plot of the mean response to LC stimulation. Strychnine increases LC responses significantly in control mice. **(D)** Plot of the mean response to vibrissal stimulation. Strychnine also increases vibrissal responses significantly in control mice. **(E)** LC-evoked Inhibition increases after STR injection. BIC applied 15 min after strychnine blocks the LC-evoked inhibition, suggesting that strychnine produces a disinhibition of GABAergic neurons since bicuculline blocks this inhibition.

Another possible candidate to inhibit vibrissal responses in the Sp5C is the glycinergic neurons that could receive axonal collaterals from LC neurons. We applied the glycinergic receptor antagonist strychnine locally into the Sp5C nucleus (100 μM; 0.2 μL) to test the participation of glycinergic neurons in the LC-evoked inhibition. The LC-evoked inhibition reduced vibrissal responses to 10.4 ± 4.0% in basal conditions and this inhibition increased up to 20.2 ± 3.8% (*p* = 0.0162; *n* = 13 neurons), 15 min after strychnine injection in control animals (Figures 4A,B). In STZ-diabetic mice, LC-evoked inhibition reduced vibrissal responses to 6.3 ± 2.7% in basal conditions and to 12.6 ± 3.8% 15 min after strychnine injection, however differences were not statistically significant (*p* > 0.05; *n* = 18 neurons). These findings indicated an increase in LC-evoked inhibition under strychnine suggesting that glycinergic neurons may inhibit GABAergic neurons, as has been suggested previously (Garcia-Magro et al., 2020).

In contrast to results observed after bicuculline injection, application of strychnine increased both LC and vibrissal orthodromic responses in control mice, suggesting that Sp5C neurons could receive a sustained, glycinergic-mediated inhibition that modulate orthodromic responses. In control animals, LC stimulation induced 2.2 ± 0.44 spikes/stimulus in basal conditions and 4.2 ± 1.1 spikes/stimulus 15 min after the injection of strychnine (*p* = 0.0245; *n* = 16 neurons; Figure 4C). Vibrissal stimulation induced 2.7 ± 0.09 spikes/stimulus in basal conditions and 4.0 ± 0.44 spikes/stimulus 15 min after the injection of strychnine (*p* = 0.0168; *n* = 17 neurons; Figure 4D). In STZ-diabetic animals, strychnine also increased orthodromic responses, however differences did not reach statistical significance. LC stimulation induced 1.2 ± 0.46 spikes/stimulus in basal conditions and 2.1 spikes/stimulus 15 min after the injection of strychnine (STR) in this animal group (*p* > 0.05; *n* = 16 neurons). Vibrissal stimulation induced 2.6 ± 0.44 spikes/stimulus in basal conditions and 2.9 ± 0.2 spikes/stimulus 15 min after the injection of strychnine (*p* > 0.05; *n* = 17).

To confirm that glycinergic neurons inhibit GABAergic neurons, we applied strychnine and 15 min later bicuculline. Figure 4E shows that STR increased LC-evoked inhibition from 8.6 ± 7.3% to 27.6 ± 6.5% (*n* = 8 neurons; *p* = 0.0434; Figure 4E, STR) due to disinhibition of GABAergic neurons while later local injection of bicuculline blocked this inhibition up to +5.5 ± 4.9% (*n* = 8 neurons; *p* = 0.0085; Figure 4E, STR + BIC). These results confirm that the activation of glycinergic receptors inhibits the activity of GABAergic neurons.

### 3.3 Paired-pulse vibrissal inhibition in control and STZ-diabetic mice

**Figure 5:**
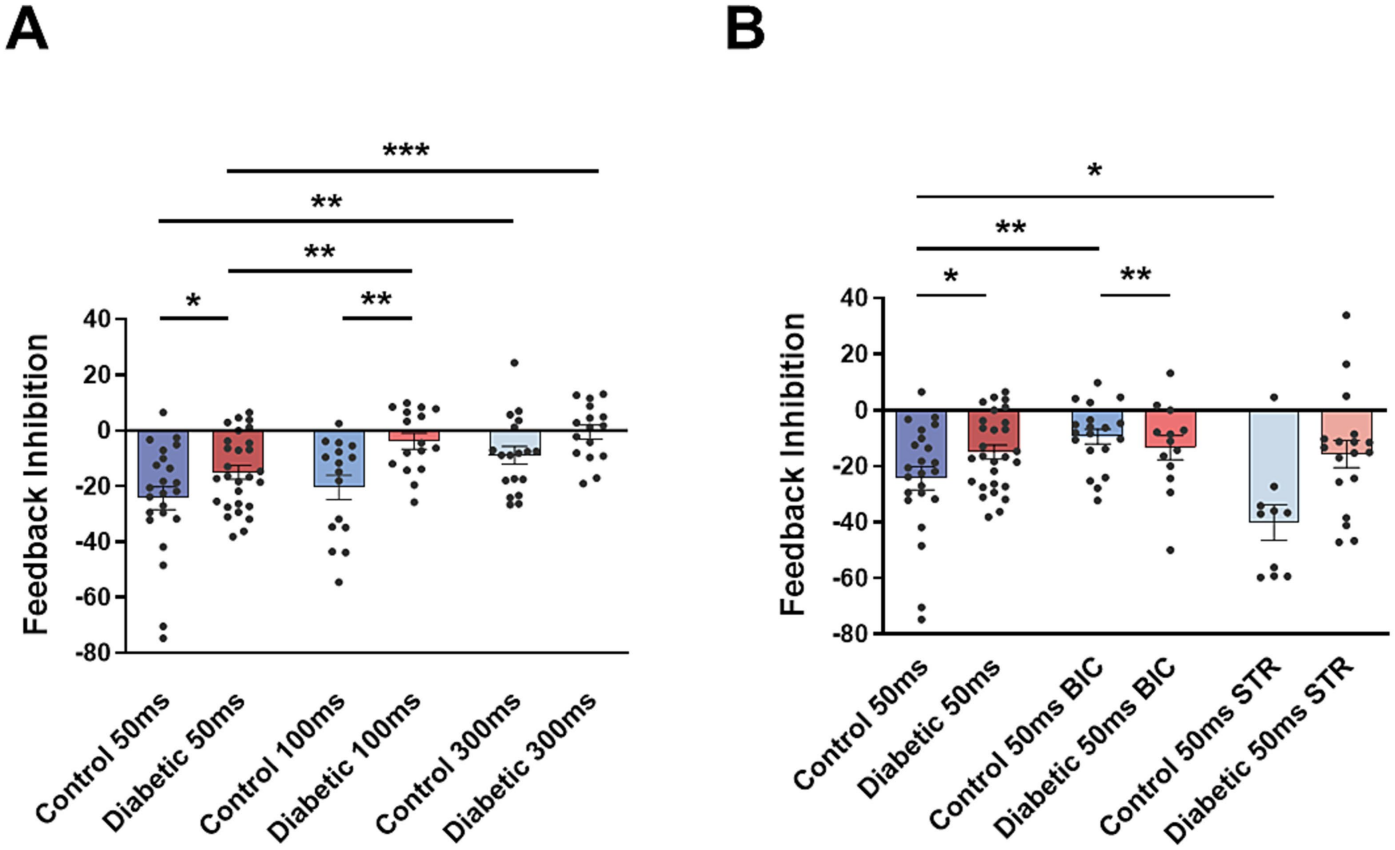
Pair-pulse inhibition of vibrissal stimuli is reduced in STZ-diabetic animals. **(A)** Plot shows the percentage of reduction of the second response when pair pulses of stimuli at the vibrissa are applied with 50, 100, and 300 ms delay (Feedback inhibition). The second response is reduced in all cases and in both animal groups. Feedback inhibition is reduced in STZ-diabetic mice in comparison with control mice. **(B)** Effect of bicuculline and strychnine on the feedback inhibition. In control animals bicuculline reduces and strychnine increases feedback inhibition. However, they have not effect in STZ-diabetic animals.

Local inhibitory circuits involving GABAergic interneurons have been proposed to participate in feedback inhibition in the sensory trigeminal nuclei, controlling sensory responses (Martin et al., 2010; Garcia-Magro et al., 2020). To test this feedback inhibition in control and STZ-diabetic mice, we applied pair pulses of stimuli at the vibrissae to quantify the reduction of the response in the second stimuli when was applied with a short delay (50, 100 and 300 ms). In control animals, paired-pulse stimulation induced a reduction of the second pulse at 50, 100 and 300 ms delays (24.3 ± 4.7%, *n* = 21 neurons; 20.4 ± 3.9%, *n* = 16 neurons; and 9 ± 3.0%, *n* = 18 neurons, respectively; Figure 5A). Paired-pulse inhibition was clearly reduced in STZ-diabetic mice. Second response was reduced to 17.15 ± 2.9%, *n* = 15 neurons; 3.8 ± 2.5%, *n* = 16 neurons; and 0.54 ± 2.2%, *n* = 16 neurons; at 50, 100 and 300 ms delay (*p* > 0.05, *p* = 0.0034 and *p* = 0.0182 respect to values in control mice).

Fifteen minutes after local injection of bicuculline in control animals, the paired-pulse inhibition (50 ms delay) was reduced from 24.8 ± 4.7%, *n* = 23 neurons, to 9.5 ± 2.7%, *n* = 19 neurons (*p* = 0.0137; Figure 5B). It was also reduced when pairs of pulses were separated by 100 ms and 300 ms in control animals (data not shown). Therefore, paired-pulse vibrissal inhibition was due to activation of GABAergic receptors. However, pair pulse inhibition was not affected by bicuculline in STZ-diabetic animals (from 17.1 ± 3.4%, *n* = 28 neurons, in basal conditions to 13.4 ± 4.5%, *n* = 13 neurons, after bicuculline application; *p* > 0.05; Figure 5B). The lack of bicuculline effect on paired-pulse inhibition in STZ-diabetic mice could indicate a reduction of GABAergic neuronal activity in these animals.

After strychnine injection in control animals, the paired-pulse inhibition at 50 ms delay increased from 24.8 ± 4.7%, *n* = 23 neurons to 40.1 ± 6.3%, *n* = 10 neurons (*p* = 0.0413; Figure 5B). Therefore, this increase after glycinergic transmission block was due to a disinhibition of GABAergic neurons. Strychnine had not effect in STZ-diabetic mice (from 17.1 ± 3.4%, *n* = 28 neurons, in basal condition to 15.7 ± 5.0%, *n* = 18 neurons; *p* > 0.05).

### 3.4 Immunohistochemistry shows the location of noradrenergic receptors in Sp5C neurons

**Figure 6:**
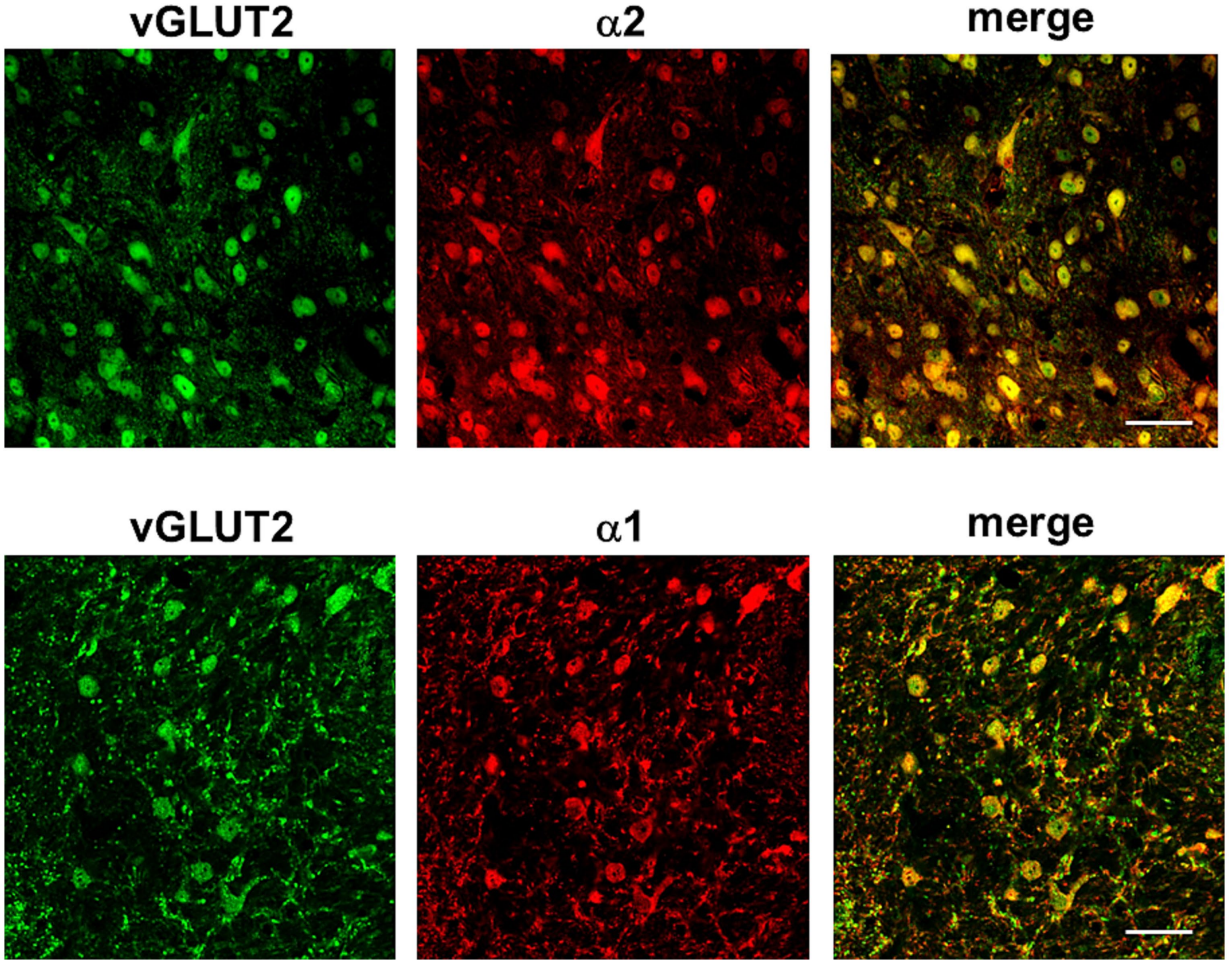
Immunohistochemistry of vGLUT2 + neurons and double staining with the α1- or α2-NA receptor antibodies. Representative photomicrographs at 63X show profuse staining with α2-NA receptors (red color, upper photomicrographs) and less with α1-NA receptors (red color, lower photomicrographs) in vGLUT2 + (green color). Scale bar 45 μm.

We have used immunohistochemical studies to show the location of NA receptors in the Sp5C neurons. The immunohistochemistry of the vesicular glutamate transporter 2 (vGLUT2), which is the protein responsible for the refilling synaptic vesicles with glutamate, showed abundant staining of cells in all laminae of the Sp5C nucleus. Double staining with the α1- or α2-NA receptor antibodies showed profuse staining in these vGLUT2 + with α2-NA receptors and much less with α1-NA receptors (*N* = 6 control mice and *N* = 6 STZ-diabetic mice; Figure 6).

**Figure 7:**
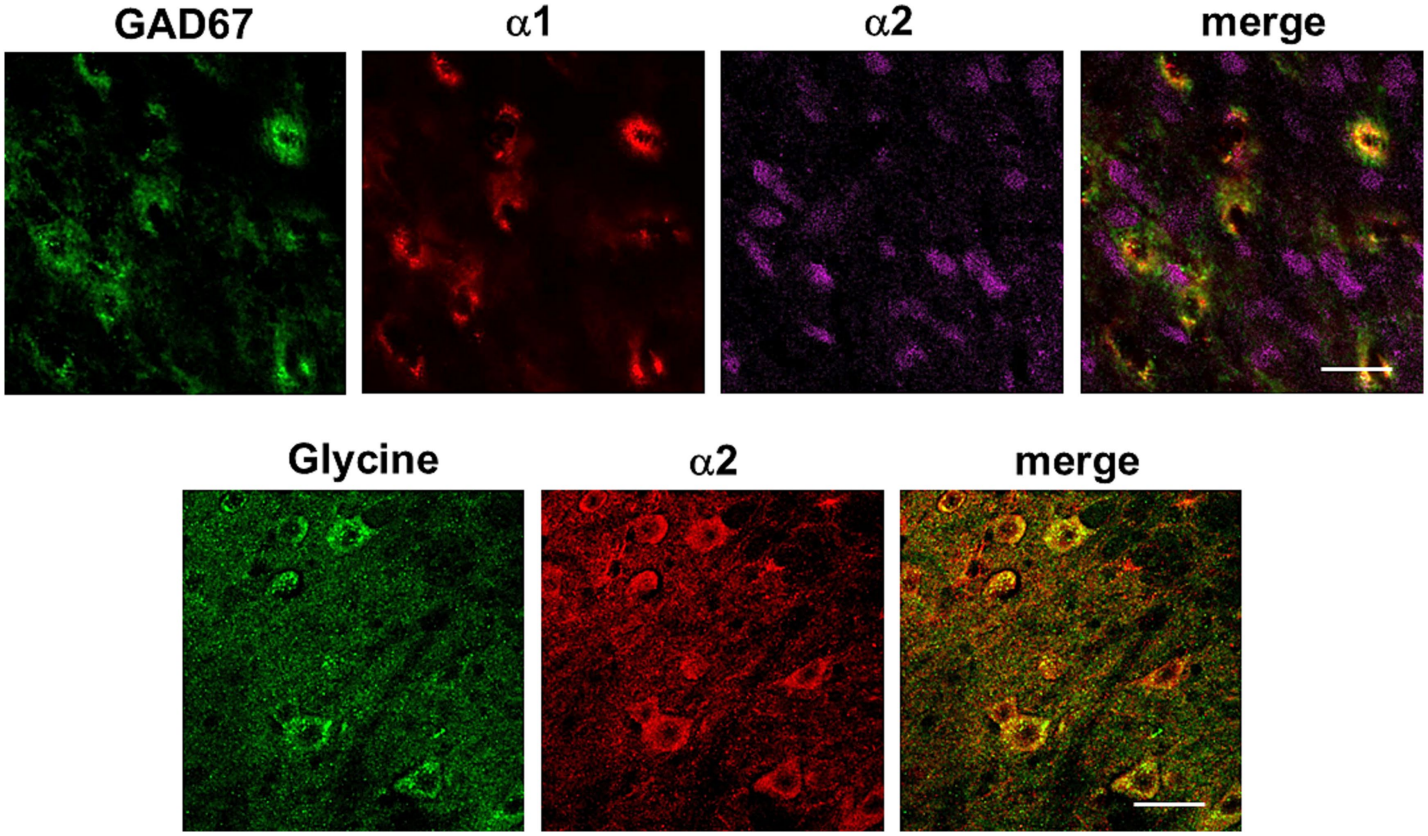
Immunohistochemistry of GABAergic neurons (GAD67), glycinergic neurons and double staining with the α1- or α2-NA receptor antibodies. Representative photomicrographs at 63X show profuse staining with α1-NA receptors (red color) but not with α2-NA receptors (magenta color) in GABAergic neurons (green color, upper photomicrographs). Lower photomicrographs show glycinergic neurons (green color) and staining with α2-NA receptor antibody (red color). Scale bar 20 μm.

On the other hand, we used glutamate decarboxylase (GAD67) antibody as a marker of GABAergic neurons. Firstly, we demonstrated that GABAergic neurons were present throughout Sp5C, with the most abundant labeling in lamina II compared to the rest of the nucleus laminae (Figure 7). Upon double staining with the α1-NA receptor antibody, we observed colocalization of both, indicating that these neurons were activated by NA through the activation of this NA receptor. Additionally, the α2-NA receptor antibody was also used, which did not colocalize with GAD67 in any case (*N* = 6 control mice and *N* = 6 STZ-diabetic mice; Figure 7).

In a separate immunohistochemical study, glycine antibody was used to stain glycinergic neurons and the α2-NA receptor antibody (*N* = 4 control mice and *N* = 4 STZ-diabetic mice; Figure 7). Co-localization was observed between the α2-NA receptor and glycinergic neurons. These experiments indicated that the α2-NA receptor was present in both glutamatergic and glycinergic neurons within the Sp5C nucleus while α1-NA receptor was present in both glutamatergic and GABAergic neurons. It is worth mentioning that there were neurons stained with α1-NA receptor antibody, which were not marked by GAD67. To our knowledge, there is no commercial antibody available to label glycinergic neurons and α1-NA receptor in the same section. Thus, we cannot rule out the possibility that neurons not labeled for these neurotransmitters (GAD67 or vGLUT2) but stained with the α1-NA receptor antibody could be glycinergic neurons.

These findings suggest a complex and distinct pattern of receptor distribution in different nuclear laminae and interaction within the neuronal networks of the Sp5C. The presence of the α2-NA receptor on both glutamatergic and glycinergic neurons and the α1 in GABAergic neurons, highlights its role in modulating the activity of diverse neuronal populations.

### 3.5 Densitometry of the immunohistochemistry between control and diabetic mice

**Figure 8:**
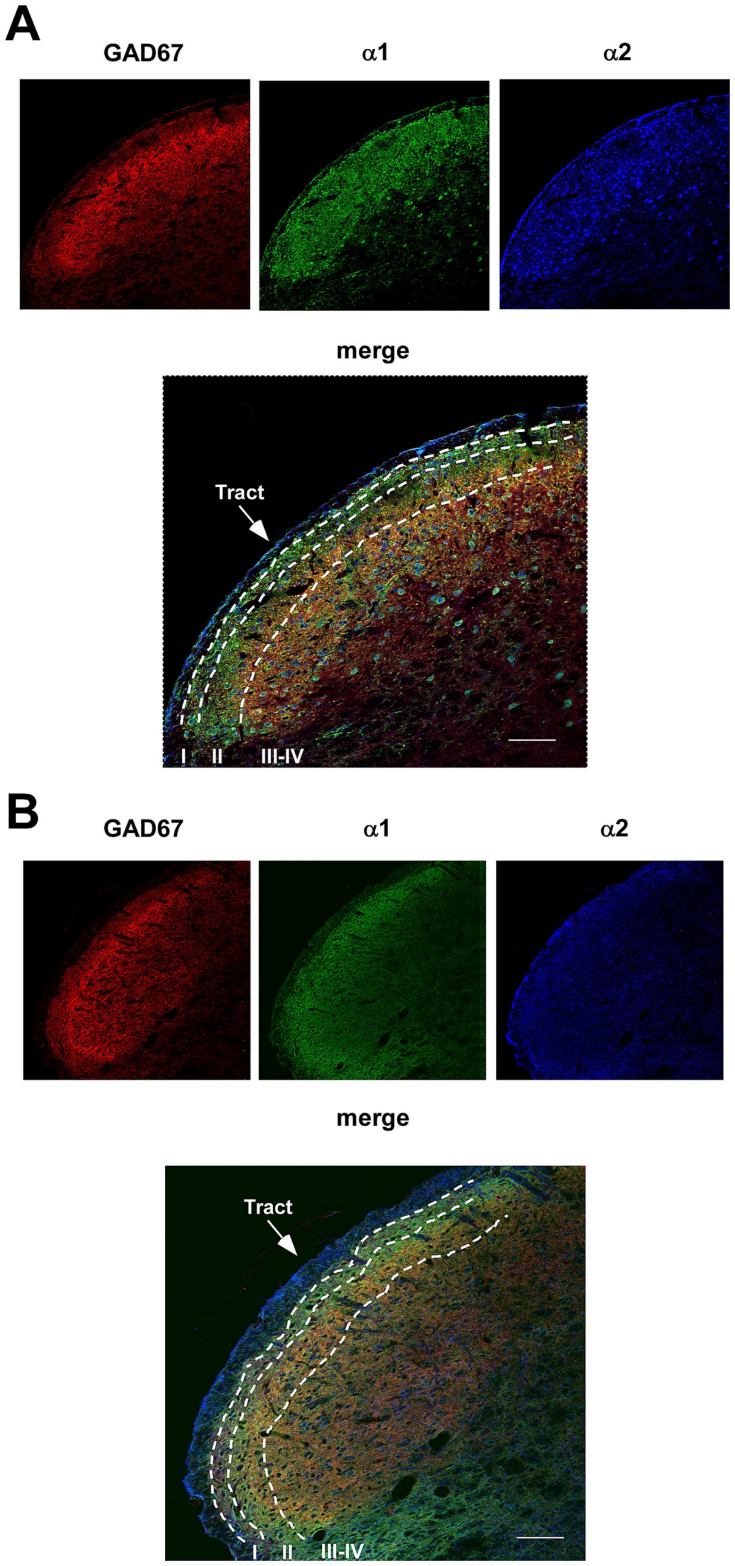
Immunohistochemistry of GABAergic neurons (GAD67) and double staining with the α1- and α2-NA receptor antibodies. Photomicrographs at 20X show all Sp5C laminae and the distribution of GABAergic neurons (red color), α1-NA receptors (green color) and α2-NA receptors (blue color) in control mice **(A)** or in STZ-diabetic mice **(B)**. GABAergic are mainly located in lamina II; α1- and α2-NA receptors are ample located in all laminae. Note that GAD67 and α2-NA receptors are reduced in STZ-diabetic mice. Scales 100 μm.

The optical density of the normalized fluorescence intensity was analyzed for GAD67, glycine, α1-, and α2-NA receptors distinguishing nuclear laminae, as is shown in Figure 8. Densitometry was performed on control animals (10 hemispheres from 6 mice, except for glycine: 8 hemispheres from 4 mice) and STZ-diabetic animals (11 hemispheres from 6 mice, except for glycine: 8 hemispheres from 4 mice) using hemispheres as the unit of analysis.

**Figure 9:**
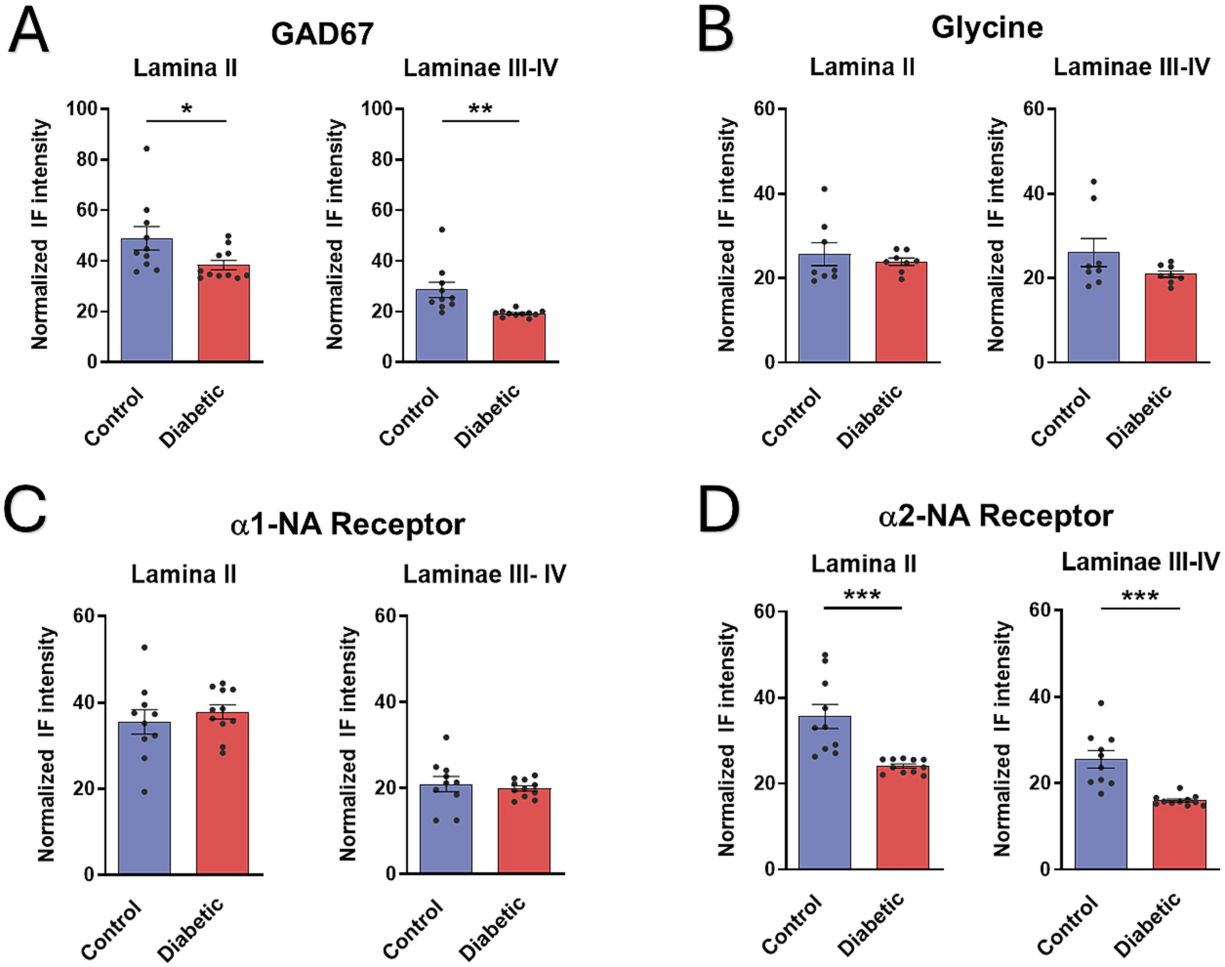
Density measures of GABAergic neurons (GAD67), glycinergic, α1- or α2-NA receptors immunoreactivity in Sp5C. **(A)** Density measures of GAD67 show statistically significant differences between control and STZ-diabetic mice in GABAergic neuron immunoreactivity in both lamina II and in laminae III-IV. **(B)** No differences are observed in glycinergic immunoreactivity in all laminae. **(C)** Density measures do not show statistically significant differences in α1-NA receptor immunoreactivity in control and STZ-diabetic mice. **(D)** Differences are observed in α2-NA receptor immunoreactivity between control and STZ-diabetic mice all laminae.

We found differences in the labeling of GAD67, being significantly higher in lamina II compared to laminae III-IV (Figure 9A). However, there were no differences in the labeling of the glycine neurotransmitter when analyzing the laminae (Figure 9B), indicating a homogeneous distribution throughout the nucleus of both animal groups. We showed a significant decrease in GAD67 labeling in STZ-diabetic mice compared to control mice, suggesting a reduction in the quantity of GABAergic neurons due to diabetes. This difference was not observed when studying glycine labeling, which remains consistent in both groups (Figures 9A,B).

Regarding the distribution of the presence of α1- and α2-NA receptors, a greater quantity of both receptors was observed in lamina II compared to laminae III-IV (Figures 9C,D). In lamina II, the quantity was similar between both NA receptors; however, a higher number of α2-NA receptors compared to α1-NA receptors was observed in laminae III-IV. This result could be attributed to a higher quantity of glutamatergic neurons throughout the nucleus (which express α2-NA receptors) compared to GABAergic neurons, which are more abundant in lamina II (which express α1-NA receptors). In addition, reduction of α2-NA receptors were observed in STZ-diabetic mice (Figure 9D), which were added to the results presented by Mesa-Lombardo et al. (2023). However, staining of α1-NA receptors remained similar between STZ-diabetic and control animals (Figure 9C).

## 4 Discussion

**Figure 10:**
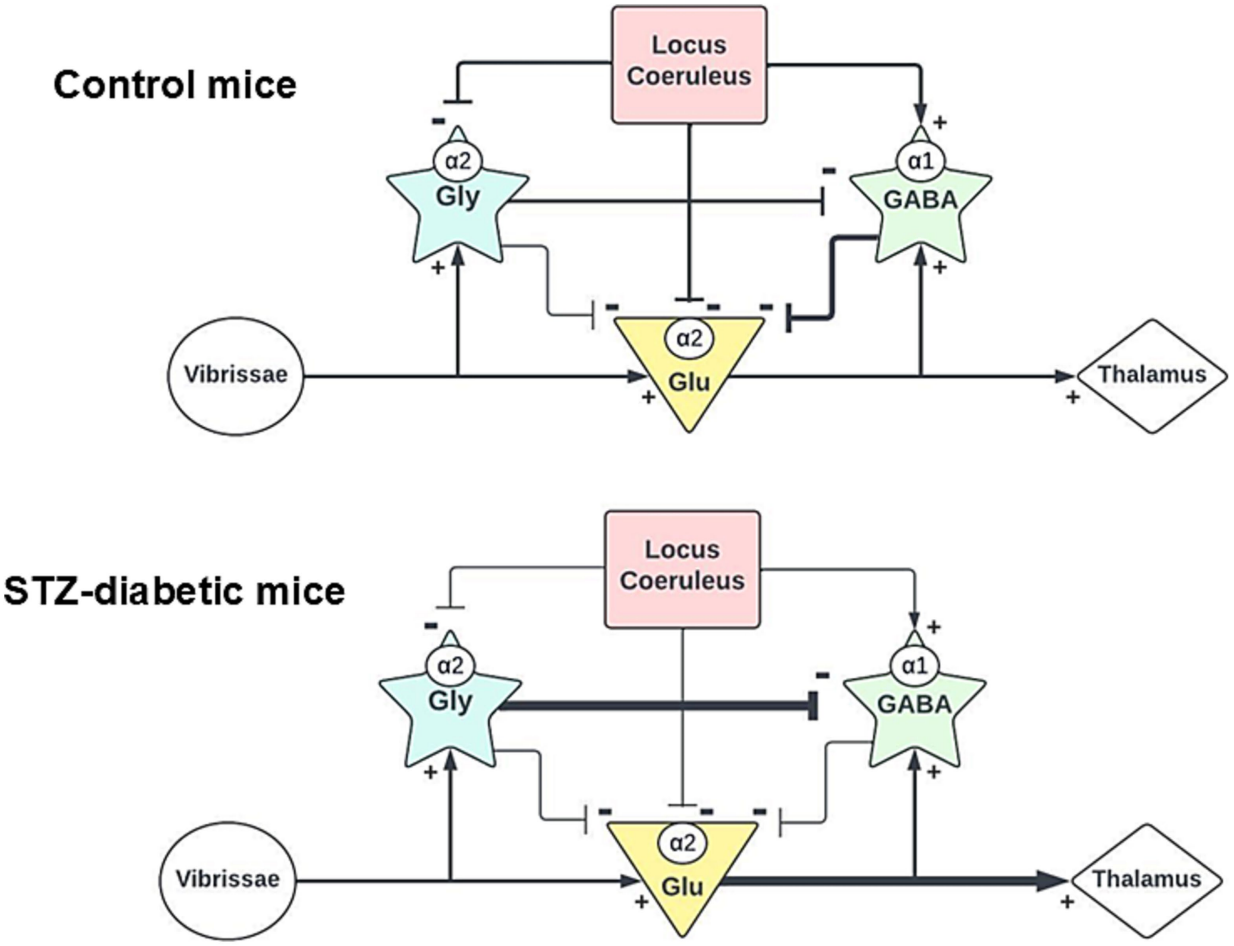
Schematic diagrams of the proposed synaptic connections in the Sp5C. Upper diagram shows that glutamatergic neurons receive inhibitory inputs from both GABA and glycinergic neurons. LC projects to all neuronal types in the Sp5C through different types of NA receptors. Our findings also show that glycinergic neurons are projected to GABAergic neurons. The Lower diagram shows the proposed changes in the synaptic connections in STZ-diabetic mice. Glycinergic connections to GABAergic neurons could increase the inhibition of GABAergic neurons and the activity of this neuronal type may be reduced.

The present study provides strong evidence for the organization and function of the LC and Sp5C network underlying the descending modulation of trigeminal sensory responses and its alterations in STZ-diabetic animals. This involves a complex synaptic interaction between NA projections and GABAergic/glycinergic neurons, acting on glutamatergic neurons. We found that NA projections on Sp5C neurons induced a direct inhibition of vibrissal responses through activation of α2-NA receptors. At the same time, GABAergic neurons were also excited by NA projections through α1-NA receptors. Therefore, the LC exerts a synergistic inhibitory effect on Sp5C neurons through direct projections and via GABAergic neurons to control sensory flow in the Sp5C nucleus. Both inhibitory effects were reduced in STZ-diabetic mice. In addition, glycinergic neurons also received NA projections from LC, which are inhibited by activation of α2-NA receptors. Our results emphasize the intricate regulatory mechanisms of the LC to regulate the sensory flow in Sp5C (Figure 10). This neuronal network that we propose may have important implications in sensory processing and for the control of chronic pain. These findings were obtained in the most common model of type 1 diabetes in rodents, the STZ-induced model. However, the mechanisms regulating sensory flow from the LC in the Sp5C shown here could be also responsible for the onset and maintenance of chronic pain in other pathologies, such as type 2 diabetes or after nerve injury.

Present experiments were performed under isoflurane anesthesia to obtain long-lasting and stable recordings in mice. It is known that isoflurane increases GABAergic transmission and impairs synaptic plasticity (Herring et al., 2009; Long Ii et al., 2016; Wang et al., 2024). However, we do not believe that our findings are due to anesthesia, considering that both experimental groups were exposed to the same anesthesia conditions; differences in synaptic plasticity must be due to the presence of diabetes since differences were also observed in immunohistochemical studies where animals were not affected by the anesthesia.

According to the spontaneous firing pattern and response characteristics of our recorded neuronal population, they should be projecting Sp5C neurons that receive inhibitory synaptic inputs from GABAergic and glycinergic neurons (Martin et al., 2010; Malmierca et al., 2012), as well as NA inputs from the LC (Pertovaara, 2013; Llorca-Torralba et al., 2016; Mesa-Lombardo et al., 2023). LC stimulation induced an orthodromic response that was not affected by α1- or α2- NA receptor antagonists. It has been published that glutamate colocalizes in most of the NA projections from the LC in rodents (Fung et al., 1994), suggesting that the short-latency orthodromic response observed after LC electrical stimulation may be due to activation of glutamatergic receptors. Indeed, the local application of the AMPA-receptor antagonist CNQX reduced the orthodromic response.

In addition, LC stimulation induced an inhibition of vibrissal responses, which was, at least in part, due to the activation of α2-NA receptors, because it was blocked by the antagonist yohimbine (Donertas-Ayaz and Caudle, 2023; Mesa-Lombardo et al., 2023) and the present results. Accordingly, the immunohistochemical results showed the presence of α2-NA receptors on glutamatergic Sp5C neurons and α1-NA receptors in a lower proportion. The α2-NA receptors induce inhibitory effects on different cell types (Jones, 1991; West et al., 1993; Sonohata et al., 2004; Han et al., 2007; Szabadi, 2013; Schwarz and Luo, 2015) through hyperpolarization of the membrane potential by G-protein-mediated activation of K + channels, as has been demonstrated in the spinal cord (Sonohata et al., 2004; Pan et al., 2008) and in the Sp5C nucleus (Han et al., 2007). Our results did not show any involvement of α1-NA receptors in the control of glutamatergic Sp5C neuronal activity although this type of receptors was observed in vGLUT2 + neurons.

Our findings indicated that LC-evoked inhibition was also due to the activation of GABAergic neurons because it was blocked by the application of the GABA_A_ receptor antagonist bicuculline. It is known that GABAergic and/or glycinergic interneurons modulate neuronal responses in sensory trigeminal nuclei (Zarbin et al., 1981; Rampon et al., 1996; Ressot et al., 2001; Bae et al., 2003; Viggiano et al., 2004; Avendaño et al., 2005; Garcia-Magro et al., 2020). TH + fibers are strongly expressed as a homogeneous band in lamina II, and more moderately in laminae III-IV (Mesa-Lombardo et al., 2023), where GABAergic neurons are located (Inquimbert et al., 2007). It is well known that laminae I and II of the Sp5C constitute the main relay station for nociception from the orofacial area (Wang et al., 2000). Thus, the concentration of TH + fibers and NA receptors in these superficial laminae suggests an important role of NA in the modulation of nociception.

The fact that LC-evoked inhibition was partially mediated by GABAergic neurons indicates that NA terminals should activate these neurons. Using immunohistochemical studies we have demonstrated the presence of α1-NA receptors on GABAergic neurons and the lack of α2-NA receptors in these neurons, which strongly suggests that the LC may increase GABAergic activity through α1-NA receptors. In general, α1-NA receptors are G protein-coupled receptors that evoke depolarization of Sp5C neurons as well as in thalamic or cortical neurons (Han et al., 2007; Perez, 2020). *In vitro* studies in the substantia gelatinosa of the guinea-pig spinal trigeminal nucleus or in the spinal cord showed that NA increased the frequency of GABA-mediated IPSPs via activation of α1-NA receptors (Grudt et al., 1995; Baba et al., 2000; Han et al., 2007). In agreement with that, LC-evoked inhibition was reduced by bicuculline, the GABA_A_ receptor antagonist, and by the α1-NA receptor antagonist benoxathian. The fact that most of the LC-evoked inhibition was blocked by bicuculline or benoxathian strongly suggests that the LC modulation of sensory responses in the Sp5C is mainly mediated by the activation of GABAergic neurons and in a low proportion by the activation of α2-NA receptors on Sp5C neurons.

Glycinergic transmission has also been described in the spinal cord and in the trigeminal complex, playing an important role in controlling sensory responses. Immunohistochemical studies have demonstrated the presence of a high density of glycine receptors and glycinergic neurons in all laminae of Sp5C (Rampon et al., 1996; Zarbin et al., 1981 and present results). Co-localization of α2-NA receptors and glycinergic neurons was observed, indicating that LC may inhibit this type of interneurons. Although we could not perform immunohistochemical staining of α1-NA receptors and glycine in the same section, we observed α1-NA receptors in neurons that were not co-stained with GAD67 or glutamatergic antibodies, suggesting that we cannot rule out the possibility that some glycinergic neurons also have α1-NA receptors. Actually, *in vitro* studies in the substantia gelatinosa of the rat spinal cord showed that NA increased the frequency of glycinergic-mediated IPSPs via activation of α1-NA (Baba et al., 2000). These findings suggest a complex and distinct pattern of NA receptor distribution and interaction within the neuronal networks of the Sp5C (Figure 10), which should be studied deeply in future experiments. The effects of NA projections may be different if the NA receptors are located on the postsynaptic cell or on the presynaptic terminal, but this type of analysis cannot be performed with our experimental setup.

We observed that strychnine increased LC-evoked inhibition or the vibrissal paired-pulse inhibition, effects that were mediated by GABAergic activity, suggesting that glycinergic terminals inhibit GABAergic neurons. In fact, laminae I–III GABAergic neurons in the spinal cord are tonically inhibited by glycine (Takazawa and MacDermott, 2010). Thus, our findings indicate the LC inhibits glutamatergic neurons by exciting GABAergic neurons through α1-NA receptors and simultaneously inhibiting glycinergic neurons through the α2-NA receptors, which in turn would disinhibit GABAergic neurons. Notably, glycinergic neurons inhibited orthodromic responses from vibrissa or LC stimulation because these synaptic responses increased under strychnine, whereas GABAergic neurons did not affect these responses (bicuculline or benoxathian did not affect these orthodromic responses). Our results suggest that there may be a tonic glycinergic inhibition on the Sp5C neurons, as occurs in the spinal cord (Takazawa and MacDermott, 2010).

It has been indicated that GABA may modulate nociceptive response in the spinal cord (Roberts et al., 1986) and in the trigeminal complex, mainly through activation of GABA_A_ receptors (Storer et al., 2001; Garcia-Magro et al., 2020). In addition, we observed that the paired-pulse inhibition mediated by GABA in the Sp5C was reduced in a chronic pain model by unilateral constriction injury of the rat infraorbital nerve (Martin et al., 2010). Our findings also suggest that GABAergic transmission may be altered in STZ-diabetic mice, facilitating the appearance of chronic pain. We found that GABAergic neurotransmission was diminished in STZ-diabetic mice because the LC-evoked inhibition and the vibrissal paired-pulse inhibition were reduced. Accordingly, the immunohistochemical staining of GAD67, the enzyme that synthesizes GABA, was reduced in all laminae of STZ-diabetic mice. Moreover, the densitometry of α2-NA receptors was also reduced in STZ-diabetic mice, indicating that the direct inhibitory action of NA terminals on Sp5C neurons was reduced. A reduction of TH + fibers was also observed in STZ-diabetic mice (Mesa-Lombardo et al., 2023 and present results). This finding may explain the reduction of the LC orthodromic response in STZ-diabetic animals compared to control animals. The fact that GAD67 was reduced in STZ-diabetic mice did not mean that GABAergic neurons disappeared. Thus, the densitometry of α1-NA receptors was equal in both control and STZ-diabetic mice. All this data together suggests that a reduction of NA inputs and GABAergic inhibitory action in the Sp5C may facilitate chronic neuropathic pain in diabetes.

## 5 Conclusion

It is firmly established that inhibitory interneurons in the Sp5C nucleus participate in the processing of sensory information. In pathological and chronic pain states, they may also play a major role contributing to central pain sensitization. Results presented here strongly suggest that GABAergic/glycinergic inhibitory interneurons are controlled by NA inputs from LC neurons. Through excitation of GABAergic neurons by α1-NA receptors or inhibition of glycinergic neurons by α2-NA receptors, the LC is capable of decreasing excitability of Sp5C neurons, which in turn would increase the threshold for transmission of noxious information. Thus, the reduction of LC activity may facilitate pain sensation in diabetes. Indeed, STZ-diabetic mice exhibit pain in the orofacial area and show a reduction in the NA fibers in the Sp5C (Mesa-Lombardo et al., 2023), which may favor the development of chronic pain in this animal model of diabetes.

Although animal models of different pathologies are generally far from the actual pathology in patients, in this case, the rodent model of diabetes using STZ is very close to real cases because this drug causes pancreatic islet *β*-cell destruction as occurs in diabetic patients. Both, animal models and diabetic patients, develop neuropathic pain and clonidine, the α2-NA receptor agonist, has been used to reduce chronic pain (Kumar et al., 2014). Our findings suggest that pharmacological treatments increasing α1-NA receptor activation or GABAergic inhibitory transmission could increase analgesic efficacy of these treatments.

## Data Availability

The raw data supporting the conclusions of this article will be made available by the authors, without undue reservation.

## References

[ref1] Alba-Delgado C. Mico J. A. Berrocoso E. (2021). Neuropathic pain increases spontaneous and noxious-evoked activity of locus coeruleus neurons. Prog. Neuro-Psychopharmacol. Biol. Psychiatry 105:110121. doi: 10.1016/j.pnpbp.2020.110121, PMID: 33007320

[ref2] Avendaño C. Machin R. Bermejo P. E. Lagares A. (2005). Neuron numbers in the sensory trigeminal nuclei of the rat: a GABA- and glycine-immunocytochemical and stereological analysis. J. Comp. Neurol. 493, 538–553. doi: 10.1002/cne.20778, PMID: 16304625

[ref3] Baba H. Shimoji K. Yoshimura M. (2000). Norepinephrine facilitates inhibitory transmission in substantia gelatinosa of adult rat spinal cord (part 1): effects on axon terminals of GABAergic and glycinergic neurons. Anesthesiology 92, 473–484. doi: 10.1097/00000542-200002000-00030 10691235

[ref4] Bae Y. C. Ihn H. J. Park M. J. Ottersen O. P. Moritani M. Yoshida A.. (2000). Identification of signal substances in synapses made between primary afferents and their associated axon terminals in the rat trigeminal sensory nuclei. J. Comp. Neurol. 418, 299–309. doi: 10.1002/(SICI)1096-9861(20000313)418:3<299::AID-CNE5>3.0.CO;2-I, PMID: 10701828

[ref5] Bae Y. C. Kim J. P. Choi B. J. Park K. P. Choi M. K. Moritani M.. (2003). Synaptic organization of tooth pulp afferent terminals in the rat trigeminal sensory nuclei. J. Comp. Neurol. 463, 13–24. doi: 10.1002/cne.10741, PMID: 12811799

[ref6] Bae Y. C. Park K. S. Bae J. Y. Paik S. K. Ahn D. K. Moritani M.. (2005). GABA and glycine in synaptic microcircuits associated with physiologically characterized primary afferents of cat trigeminal principal nucleus. Exp. Brain Res. 162, 449–457. doi: 10.1007/s00221-004-2022-y, PMID: 15678357

[ref7] Basbaum A. I. Glazer E. J. Oertel W. (1986). Immunoreactive glutamic acid decarboxylase in the trigeminal nucleus caudalis of the cat: a light- and electron-microscopic analysis. Somatosens. Res. 4, 77–94. doi: 10.3109/07367228609144599, PMID: 3541116

[ref8] Benarroch E. E. (2018). Locus coeruleus. Cell Tissue Res. 373, 221–232. doi: 10.1007/s00441-017-2649-1, PMID: 28687925

[ref9] Bereiter D. A. Hirata H. Hu J. W. (2000). Trigeminal subnucleus caudalis: beyond homologies with the spinal dorsal horn. Pain 88, 221–224. doi: 10.1016/S0304-3959(00)00434-6, PMID: 11068108

[ref10] Berridge C. W. Foote S. L. (1991). Effects of locus coeruleus activation on electroencephalographic activity in neocortex and hippocampus. J. Neurosci. 11, 3135–3145. doi: 10.1523/JNEUROSCI.11-10-03135.1991, PMID: 1682425 PMC3058938

[ref11] Borodovitsyna O. Flamini M. Chandler D. (2017). Noradrenergic modulation of cognition in health and disease. Neural Plast. 2017, 6031478–6031414. doi: 10.1155/2017/6031478, PMID: 28596922 PMC5450174

[ref12] Brightwell J. J. Taylor B. K. (2009). Noradrenergic neurons in the locus coeruleus contribute to neuropathic pain. Neuroscience 160, 174–185. doi: 10.1016/j.neuroscience.2009.02.023, PMID: 19223010 PMC2677992

[ref13] Couto L. B. Moroni C. R. dos Reis Ferreira C. M. Elias-Filho D. H. Parada C. A. Pela I. R.. (2006). Descriptive and functional neuroanatomy of locus coeruleus-noradrenaline-containing neurons involvement in bradykinin-induced antinociception on principal sensory trigeminal nucleus. J. Chem. Neuroanat. 32, 28–45. doi: 10.1016/j.jchemneu.2006.03.003, PMID: 16678997

[ref14] Crawley J. N. Roth R. H. Maas J. W. (1979). Locus coeruleus stimulation increases noradrenergic metabolite levels in rat spinal cord. Brain Res. 166, 180–184. doi: 10.1016/0006-8993(79)90661-9, PMID: 421150

[ref15] Cropper E. C. Eisenman J. S. Azmitia E. C. (1984). 5-HT-immunoreactive fibers in the trigeminal nuclear complex of the rat. Exp. Brain Res. 55, 515–522. doi: 10.1007/BF00235282, PMID: 6381083

[ref16] Devilbiss D. M. Page M. E. Waterhouse B. D. (2006). Locus ceruleus regulates sensory encoding by neurons and networks in waking animals. J. Neurosci. 26, 9860–9872. doi: 10.1523/JNEUROSCI.1776-06.2006, PMID: 17005850 PMC6674489

[ref17] Devilbiss D. M. Waterhouse B. D. (2004). The effects of tonic locus ceruleus output on sensory-evoked responses of ventral posterior medial thalamic and barrel field cortical neurons in the awake rat. J. Neurosci. 24, 10773–10785. doi: 10.1523/JNEUROSCI.1573-04.2004, PMID: 15574728 PMC6730210

[ref18] Dickenson A. H. Hellon R. F. Woolf C. J. (1981). Tooth pulp input to the spinal trigeminal nucleus: a comparison of inhibitions following segmental and raphe magnus stimulation. Brain Res. 214, 73–87. doi: 10.1016/0006-8993(81)90439-x, PMID: 7237167

[ref19] Donertas-Ayaz B. Caudle R. M. (2023). Locus coeruleus-noradrenergic modulation of trigeminal pain: implications for trigeminal neuralgia and psychiatric comorbidities. Neurobiol. Pain 13:100124. doi: 10.1016/j.ynpai.2023.100124, PMID: 36974102 PMC10038791

[ref20] España J. C. Yasoda-Mohan A. Vanneste S. (2024). The locus Coeruleus in chronic pain. Int. J. Mol. Sci. 25:8636. doi: 10.3390/ijms25168636, PMID: 39201323 PMC11354431

[ref21] Foote S. L. Loughlin S. E. Cohen P. S. Bloom F. E. Livingston R. B. (1980). Accurate three-dimensional reconstruction of neuronal distributions in brain: reconstruction of the rat nucleus locus coeruleus. J. Neurosci. Methods 3, 159–173. doi: 10.1016/0165-0270(80)90023-0, PMID: 7206782

[ref22] Fung S. J. Reddy V. K. Liu R. H. Wang Z. Barnes C. D. (1994). Existence of glutamate in noradrenergic locus coeruleus neurons of rodents. Brain Res. Bull. 35, 505–512. doi: 10.1016/0361-9230(94)90164-3, PMID: 7859108

[ref23] Furman B. L. (2021). Streptozotocin-induced diabetic models in mice and rats. Curr. Protoc. 1:e78. doi: 10.1002/cpz1.78, PMID: 33905609

[ref24] Garcia-Magro N. Martin Y. B. Negredo P. Zafra F. Avendano C. (2021). Microglia and inhibitory circuitry in the medullary dorsal horn: laminar and time-dependent changes in a trigeminal model of neuropathic pain. Int. J. Mol. Sci. 22:4564. doi: 10.3390/ijms22094564, PMID: 33925417 PMC8123867

[ref25] Garcia-Magro N. Negredo P. Martin Y. B. Nunez A. Avendano C. (2020). Modulation of mechanosensory vibrissal responses in the trigeminocervical complex by stimulation of the greater occipital nerve in a rat model of trigeminal neuropathic pain. J. Headache Pain 21:96. doi: 10.1186/s10194-020-01161-y, PMID: 32762640 PMC7410158

[ref26] Glavin G. B. (1985). Stress and brain noradrenaline: a review. Neurosci. Biobehav. Rev. 9, 233–243. doi: 10.1016/0149-7634(85)90048-x, PMID: 2861590

[ref001] Grudt T. J. Williams J. T. Travagli R. A. (1995). Inhibition by 5‐hydroxytryptamine and noradrenaline in substantia gelatinosa of guinea‐pig spinal trigeminal nucleus. J. Physiol. 485, 113–120. doi: 10.1113/jphysiol.1995.sp020716, PMID: 7658366 PMC1157976

[ref27] Han S. K. Park J. R. Park S. A. Chun S. W. Lee J. C. Lee S. Y.. (2007). Noradrenaline inhibits substantia gelatinosa neurons in mice trigeminal subnucleus caudalis via alpha(2) and beta adrenoceptors. Neurosci. Lett. 411, 92–97. doi: 10.1016/j.neulet.2006.10.041 17110030

[ref28] Herring B. E. Xie Z. Marks J. Fox A. P. (2009). Isoflurane inhibits the neurotransmitter release machinery. J. Neurophysiol. 102, 1265–1273. doi: 10.1152/jn.00252.2009, PMID: 19515956 PMC2724342

[ref29] Hickey L. Li Y. Fyson S. J. Watson T. C. Perrins R. Hewinson J.. (2014). Optoactivation of locus ceruleus neurons evokes bidirectional changes in thermal nociception in rats. J. Neurosci. 34, 4148–4160. doi: 10.1523/JNEUROSCI.4835-13.2014, PMID: 24647936 PMC3960461

[ref30] Hirata A. Aguilar J. Castro-Alamancos M. A. (2006). Noradrenergic activation amplifies bottom-up and top-down signal-to-noise ratios in sensory thalamus. J. Neurosci. 26, 4426–4436. doi: 10.1523/JNEUROSCI.5298-05.2006, PMID: 16624962 PMC6674001

[ref31] Hodge C. J. Jr. Apkarian A. V. Stevens R. Vogelsang G. Wisnicki H. J. (1981). Locus coeruleus modulation of dorsal horn unit responses to cutaneous stimulation. Brain Res. 204, 415–420. doi: 10.1016/0006-8993(81)90600-4, PMID: 6257328

[ref32] Hu J. W. (1990). Response properties of nociceptive and non-nociceptive neurons in the rat's trigeminal subnucleus caudalis (medullary dorsal horn) related to cutaneous and deep craniofacial afferent stimulation and modulation by diffuse noxious inhibitory controls. Pain 41, 331–345. doi: 10.1016/0304-3959(90)90010-B, PMID: 2388770

[ref33] Inquimbert P. Rodeau J. L. Schlichter R. (2007). Differential contribution of GABAergic and glycinergic components to inhibitory synaptic transmission in lamina II and laminae III-IV of the young rat spinal cord. Eur. J. Neurosci. 26, 2940–2949. doi: 10.1111/j.1460-9568.2007.05919.x, PMID: 18001289

[ref34] Jacquin M. F. Golden J. Rhoades R. W. (1989). Structure-function relationships in rat brainstem subnucleus interpolaris: III. Local circuit neurons. J. Comp. Neurol. 282, 24–44. doi: 10.1002/cne.902820104, PMID: 2708592

[ref35] Jacquin M. F. Renehan W. E. Mooney R. D. Rhoades R. W. (1986). Structure-function relationships in rat medullary and cervical dorsal horns. I. Trigeminal primary afferents. J. Neurophysiol. 55, 1153–1186. doi: 10.1152/jn.1986.55.6.1153, PMID: 3734853

[ref36] Jones S. L. (1991). Descending noradrenergic influences on pain. Prog. Brain Res. 88, 381–394. doi: 10.1016/s0079-6123(08)63824-8, PMID: 1813927

[ref37] Jones S. L. Gebhart G. F. (1986). Quantitative characterization of ceruleospinal inhibition of nociceptive transmission in the rat. J. Neurophysiol. 56, 1397–1410. doi: 10.1152/jn.1986.56.5.1397, PMID: 3025380

[ref38] Kumar A. Maitra S. Khanna P. Baidya D. K. (2014). Clonidine for management of chronic pain: a brief review of the current evidences. Saudi J Anaesth 8, 92–96. doi: 10.4103/1658-354X.125955, PMID: 24665248 PMC3950462

[ref39] Levitt P. Moore R. Y. (1979). Origin and organization of brainstem catecholamine innervation in the rat. J. Comp. Neurol. 186, 505–528. doi: 10.1002/cne.901860402, PMID: 15116686

[ref40] Llorca-Torralba M. Borges G. Neto F. Mico J. A. Berrocoso E. (2016). Noradrenergic locus coeruleus pathways in pain modulation. Neuroscience 338, 93–113. doi: 10.1016/j.neuroscience.2016.05.057, PMID: 27267247

[ref41] Long Ii R. P. Aroniadou-Anderjaska V. Prager E. M. Pidoplichko V. I. Figueiredo T. H. Braga M. F. (2016). Repeated isoflurane exposures impair Long-term potentiation and increase basal GABAergic activity in the basolateral amygdala. Neural Plast. 2016, 8524560–8524569. doi: 10.1155/2016/8524560, PMID: 27313904 PMC4893574

[ref42] Loughlin S. E. Foote S. L. Bloom F. E. (1986). Efferent projections of nucleus locus coeruleus: topographic organization of cells of origin demonstrated by three-dimensional reconstruction. Neuroscience 18, 291–306. doi: 10.1016/0306-4522(86)90155-7, PMID: 3736860

[ref43] Malmierca E. Martin Y. B. Nuñez A. (2012). Inhibitory control of nociceptive responses of trigeminal spinal nucleus cells by somatosensory corticofugal projection in rat. Neuroscience 221, 115–124. doi: 10.1016/j.neuroscience.2012.07.003, PMID: 22796078

[ref44] Manella L. C. Petersen N. Linster C. (2017). Stimulation of the locus Ceruleus modulates signal-to-noise ratio in the olfactory bulb. J. Neurosci. 37, 11605–11615. doi: 10.1523/JNEUROSCI.2026-17.2017, PMID: 29066553 PMC5707764

[ref45] Marfurt C. F. Turner D. F. (1984). The central projections of tooth pulp afferent neurons in the rat as determined by the transganglionic transport of horseradish peroxidase. J. Comp. Neurol. 223, 535–547. doi: 10.1002/cne.902230406, PMID: 6325510

[ref46] Martin Y. B. Malmierca E. Avendaño C. Nuñez A. (2010). Neuronal disinhibition in the trigeminal nucleus caudalis in a model of chronic neuropathic pain. Eur. J. Neurosci. 32, 399–408. doi: 10.1111/j.1460-9568.2010.07302.x, PMID: 20704591

[ref47] McBride R. L. Sutin J. (1984). Noradrenergic hyperinnervation of the trigeminal sensory nuclei. Brain Res. 324, 211–221. doi: 10.1016/0006-8993(84)90031-3, PMID: 6099202

[ref48] Mesa-Lombardo A. Garcia-Magro N. Nunez A. Martin Y. B. (2023). Locus coeruleus inhibition of vibrissal responses in the trigeminal subnucleus caudalis are reduced in a diabetic mouse model. Front. Cell. Neurosci. 17:1208121. doi: 10.3389/fncel.2023.1208121, PMID: 37475984 PMC10354250

[ref49] Mitchell H. A. Weinshenker D. (2010). Good night and good luck: norepinephrine in sleep pharmacology. Biochem. Pharmacol. 79, 801–809. doi: 10.1016/j.bcp.2009.10.004, PMID: 19833104 PMC2812689

[ref50] Orstavik K. Namer B. Schmidt R. Schmelz M. Hilliges M. Weidner C.. (2006). Abnormal function of C-fibers in patients with diabetic neuropathy. J. Neurosci. 26, 11287–11294. doi: 10.1523/JNEUROSCI.2659-06.2006, PMID: 17079656 PMC6674548

[ref51] Pan H. L. Wu Z. Z. Zhou H. Y. Chen S. R. Zhang H. M. Li D. P. (2008). Modulation of pain transmission by G-protein-coupled receptors. Pharmacol. Ther. 117, 141–161. doi: 10.1016/j.pharmthera.2007.09.003, PMID: 17959251 PMC2965406

[ref52] Paxinos G. Franklin K. B. J. (2003). The mouse brain in stereotaxic coordinates. San Diego, CA: Academic Press.

[ref53] Perez D. M. (2020). Alpha(1)-adrenergic receptors in neurotransmission, synaptic plasticity, and cognition. Front. Pharmacol. 11:581098. doi: 10.3389/fphar.2020.581098 33117176 PMC7553051

[ref54] Pertovaara A. (2013). The noradrenergic pain regulation system: a potential target for pain therapy. Eur. J. Pharmacol. 716, 2–7. doi: 10.1016/j.ejphar.2013.01.067 23500194

[ref55] Priestley J. V. Somogyi P. Cuello A. C. (1982). Immunocytochemical localization of substance P in the spinal trigeminal nucleus of the rat: a light and electron microscopic study. J. Comp. Neurol. 211, 31–49. doi: 10.1002/cne.902110105, PMID: 6184386

[ref56] Rampon C. Luppi P. H. Fort P. Peyron C. Jouvet M. (1996). Distribution of glycine-immunoreactive cell bodies and fibers in the rat brain. Neuroscience 75, 737–755. doi: 10.1016/0306-4522(96)00278-3, PMID: 8951870

[ref57] Ressot C. Collado V. Molat J. L. Dallel R. (2001). Strychnine alters response properties of trigeminal nociceptive neurons in the rat. J. Neurophysiol. 86, 3069–3072. doi: 10.1152/jn.2001.86.6.3069, PMID: 11731563

[ref58] Rho H. J. Kim J. H. Lee S. H. (2018). Function of selective Neuromodulatory projections in the mammalian cerebral cortex: comparison between cholinergic and noradrenergic systems. Front. Neural Circuits 12:47. doi: 10.3389/fncir.2018.00047, PMID: 29988373 PMC6023998

[ref002] Roberts L. A. Beyer C. Komisaruk B. R. (1986). Nociceptive responses to altered GABAergic activity at the spinal cord. Life sciences, 39:1667–1674. doi: 10.1016/0024-3205(86)90164-5, PMID: 3022091

[ref59] Sasa M. Takaori S. (1973). Influence of the locus coeruleus on transmission in the spinal trigeminal nucleus neurons. Brain Res. 55, 203–208. doi: 10.1016/0006-8993(73)90502-7, PMID: 4351518

[ref60] Schwarz L. A. Luo L. (2015). Organization of the locus coeruleus-norepinephrine system. Curr. Biol. 25, R1051–R1056. doi: 10.1016/j.cub.2015.09.039, PMID: 26528750

[ref61] Shinoda M. Kubo A. Hayashi Y. Iwata K. (2019). Peripheral and central mechanisms of persistent orofacial pain. Front. Neurosci. 13:1227. doi: 10.3389/fnins.2019.01227, PMID: 31798407 PMC6863776

[ref62] Sivakumar D. Ramli R. (2022). GABAergic signalling in modulation of dental pain. Eur. J. Pharmacol. 924:174958. doi: 10.1016/j.ejphar.2022.174958, PMID: 35429491

[ref63] Sonohata M. Furue H. Katafuchi T. Yasaka T. Doi A. Kumamoto E.. (2004). Actions of noradrenaline on substantia gelatinosa neurones in the rat spinal cord revealed by in vivo patch recording. J. Physiol. 555, 515–526. doi: 10.1113/jphysiol.2003.054932, PMID: 14673188 PMC1664849

[ref64] Storer R. J. Akerman S. Goadsby P. J. (2001). GABA receptors modulate trigeminovascular nociceptive neurotransmission in the trigeminocervical complex. Br. J. Pharmacol. 134, 896–904. doi: 10.1038/sj.bjp.0704325, PMID: 11606331 PMC1573015

[ref65] Suzuki C. Ozaki I. Tanosaki M. Suda T. Baba M. Matsunaga M. (2000). Peripheral and central conduction abnormalities in diabetes mellitus. Neurology 54, 1932–1937. doi: 10.1212/WNL.54.10.1932, PMID: 10822432

[ref66] Szabadi E. (2013). Functional neuroanatomy of the central noradrenergic system. J. Psychopharmacol. 27, 659–693. doi: 10.1177/0269881113490326, PMID: 23761387

[ref67] Takazawa T. MacDermott A. B. (2010). Glycinergic and GABAergic tonic inhibition fine tune inhibitory control in regionally distinct subpopulations of dorsal horn neurons. J. Physiol. 588, 2571–2587. doi: 10.1113/jphysiol.2010.188292, PMID: 20498232 PMC2916989

[ref68] Takemura M. Sugiyo S. Moritani M. Kobayashi M. Yonehara N. (2006). Mechanisms of orofacial pain control in the central nervous system. Arch. Histol. Cytol. 69, 79–100. doi: 10.1679/aohc.69.79, PMID: 16819148

[ref69] Terrier L. M. Hadjikhani N. Destrieux C. (2022). The trigeminal pathways. J. Neurol. 269, 3443–3460. doi: 10.1007/s00415-022-11002-4, PMID: 35249132

[ref70] Tsuruoka M. Matsutani K. Inoue T. (2003a). Coeruleospinal inhibition of nociceptive processing in the dorsal horn during unilateral hindpaw inflammation in the rat. Pain 104, 353–361. doi: 10.1016/s0304-3959(03)00042-3, PMID: 12855345

[ref71] Tsuruoka M. Matsutani K. Maeda M. Inoue T. (2003b). Coeruleotrigeminal inhibition of nociceptive processing in the rat trigeminal subnucleus caudalis. Brain Res. 993, 146–153. doi: 10.1016/j.brainres.2003.09.023, PMID: 14642840

[ref72] Tsuruoka M. Willis W. D. (1996). Descending modulation from the region of the locus coeruleus on nociceptive sensitivity in a rat model of inflammatory hyperalgesia. Brain Res. 743, 86–92. doi: 10.1016/s0006-8993(96)01025-6, PMID: 9017234

[ref73] Valentino R. J. Foote S. L. Page M. E. (1993). The locus coeruleus as a site for integrating corticotropin-releasing factor and noradrenergic mediation of stress responses. Ann. N. Y. Acad. Sci. 697, 173–188. doi: 10.1111/j.1749-6632.1993.tb49931.x, PMID: 7903030

[ref74] Viggiano A. Monda M. Viggiano A. Chiefari M. Aurilio C. De Luca B. (2004). Evidence that GABAergic neurons in the spinal trigeminal nucleus are involved in the transmission of inflammatory pain in the rat: a microdialysis and pharmacological study. Eur. J. Pharmacol. 496, 87–92. doi: 10.1016/j.ejphar.2004.06.019, PMID: 15288579

[ref75] Wang Z. Chen K. Wu X. Zheng P. Li A. Guo Y.. (2024). Distinct neural activities of the cortical layer 2/3 across isoflurane anesthesia: a large-scale simultaneous observation of neurons. Biomed. Pharmacother. 175:116751. doi: 10.1016/j.biopha.2024.116751, PMID: 38754266

[ref76] Wang D. Li Y. Q. Li J. L. Kaneko T. Nomura S. Mizuno N. (2000). Gamma-aminobutyric acid- and glycine-immunoreactive neurons postsynaptic to substance P-immunoreactive axon terminals in the superficial layers of the rat medullary dorsal horn. Neurosci. Lett. 288, 187–190. doi: 10.1016/s0304-3940(00)01226-x, PMID: 10889339

[ref77] Waterhouse B. D. Moises H. C. Woodward D. J. (1998). Phasic activation of the locus coeruleus enhances responses of primary sensory cortical neurons to peripheral receptive field stimulation. Brain Res. 790, 33–44. doi: 10.1016/S0006-8993(98)00117-6, PMID: 9593812

[ref78] West W. L. Yeomans D. C. Proudfit H. K. (1993). The function of noradrenergic neurons in mediating antinociception induced by electrical stimulation of the locus coeruleus in two different sources of Sprague-Dawley rats. Brain Res. 626, 127–135. doi: 10.1016/0006-8993(93)90571-4, PMID: 7904225

[ref79] Zarbin M. A. Wamsley J. K. Kuhar M. J. (1981). Glycine receptor: light microscopic autoradiographic localization with [3H]strychnine. J. Neurosci. 1, 532–547. doi: 10.1523/JNEUROSCI.01-05-00532.1981, PMID: 6286895 PMC6564171

[ref80] Zeilhofer H. U. Werynska K. Gingras J. Yevenes G. E. (2021). Glycine receptors in spinal nociceptive control-an update. Biomol. Ther. 11:846. doi: 10.3390/biom11060846, PMID: 34204137 PMC8228028

